# Long-term antigen reduction does not achieve durable HBV control in mice but enhances therapeutic vaccine efficacy

**DOI:** 10.1016/j.jhepr.2026.101943

**Published:** 2026-06-30

**Authors:** Thomas Michler, Anna Kosinska, Osman Merdan, Philipp Hagen, Till Bunse, Edanur Ates Öz, Jinpeng Su, Dirk H. Busch, Carolin Mogler, Ulrike Protzer

**Affiliations:** 1 Institute of Virology, School of Medicine and Health, Technical University of Munich/Helmholtz, Munich, Germany; 2 Institute of Laboratory Medicine, LMU University Hospital, LMU Munich, Munich, Germany; 3 German Center for Infection Research (DZIF), Munich partner site, Munich, Germany; 4 Institute of Medical Microbiology, Immunology and Hygiene, School of Medicine and Health, Technical University of Munich, Munich, Germany; 5 Institute of Pathology, School of Medicine and Health, Technical University of Munich, Munich, Germany

**Keywords:** Chronic hepatitis B, HBV cure, Immunotolerance, Therapeutic vaccination, siRNA, Combination therapy

## Abstract

**Background & Aims:**

Chronic HBV infection has an increasing death toll, but curative therapies are lacking. The study aimed to determine whether long-term suppression of viral antigens using RNA-interference restores HBV-specific immunity and achieves durable virus control, and how long HBV antigens must be suppressed to enable therapeutic vaccination to restore immunity.

**Methods:**

HBV-transgenic or AAV-HBV-infected, HBV-carrier mice were treated for up to 7 months with liver-directed small interfering RNAs (siRNAs) or short-hairpin RNAs (shRNAs) that target all HBV transcripts. The antiviral effect and development of B- and T-cell immunity were evaluated. A subcohort of mice received the heterologous prime/boost therapeutic vaccine, *TherVacB*, before cessation of siRNA.

**Results:**

Continuous siRNA therapy reduced HBsAg by up to 4 log_10_, but a threshold effect was observed after 12–16 weeks. Suppression of viral antigens for up to 7 months did not lead to spontaneous reconstitution of T-cell immunity; this required stimulation by therapeutic vaccination. Without vaccination, HBV replication rebounded after discontinuation of siRNA therapy, even though HBsAg and HBeAg seroconversion had occurred. However, a longer duration of siRNA pretreatment correlated with increasing interferon type I signaling in the liver. siRNA pretreatment for 8 instead of 3.5 weeks improved the ability of therapeutic vaccination to activate functional antiviral T cells, fully control HBV replication, and significantly reduce the number of HBV-positive hepatocytes (*p* <0.01 to *p* <0.001).

**Conclusions:**

Our data show that, in addition to suppressing viral antigens, immune stimulation is necessary to achieve long-lasting HBV control after treatment discontinuation. These results will help design clinical trials that can ultimately achieve HBV control.

**Impact and implications:**

Drug entities in development to cure chronic hepatitis B include direct-acting antivirals, nucleic acid-based therapeutics, immunotherapies, and combinations thereof. In our study, long-term suppression of HBV in HBV-carrier mice using siRNAs targeting all HBV antigens neither allowed spontaneous reconstitution of T-cell immunity nor sustained HBV control, even when accompanied by anti-HBs seroconversion. However, prolonged viral antigen suppression by siRNA enhanced the efficacy of therapeutic vaccination. Our study highlights that achieving loss of viral parameters from serum, or even anti-HBe/HBs seroconversion, should not represent the ultimate goal of curative therapy, but rather be viewed as a prerequisite that empowers immunostimulatory drugs to induce curative T-cell responses.

## Highlights

• Long-term suppression of HBV by siRNA did not enable sustained HBV control.
• Suppression of HBsAg and HBeAg to undetectable levels did not restore antiviral immunity.
• Therapeutic vaccination was necessary to achieve HBV control after cessation of siRNA therapy.
• Kinetics of viral antigens in the liver correlated best with the success of immune restoration.
• A longer HBV suppression by siRNA enhanced the efficacy of therapeutic vaccination.

## Graphical abstract

**Figure:**
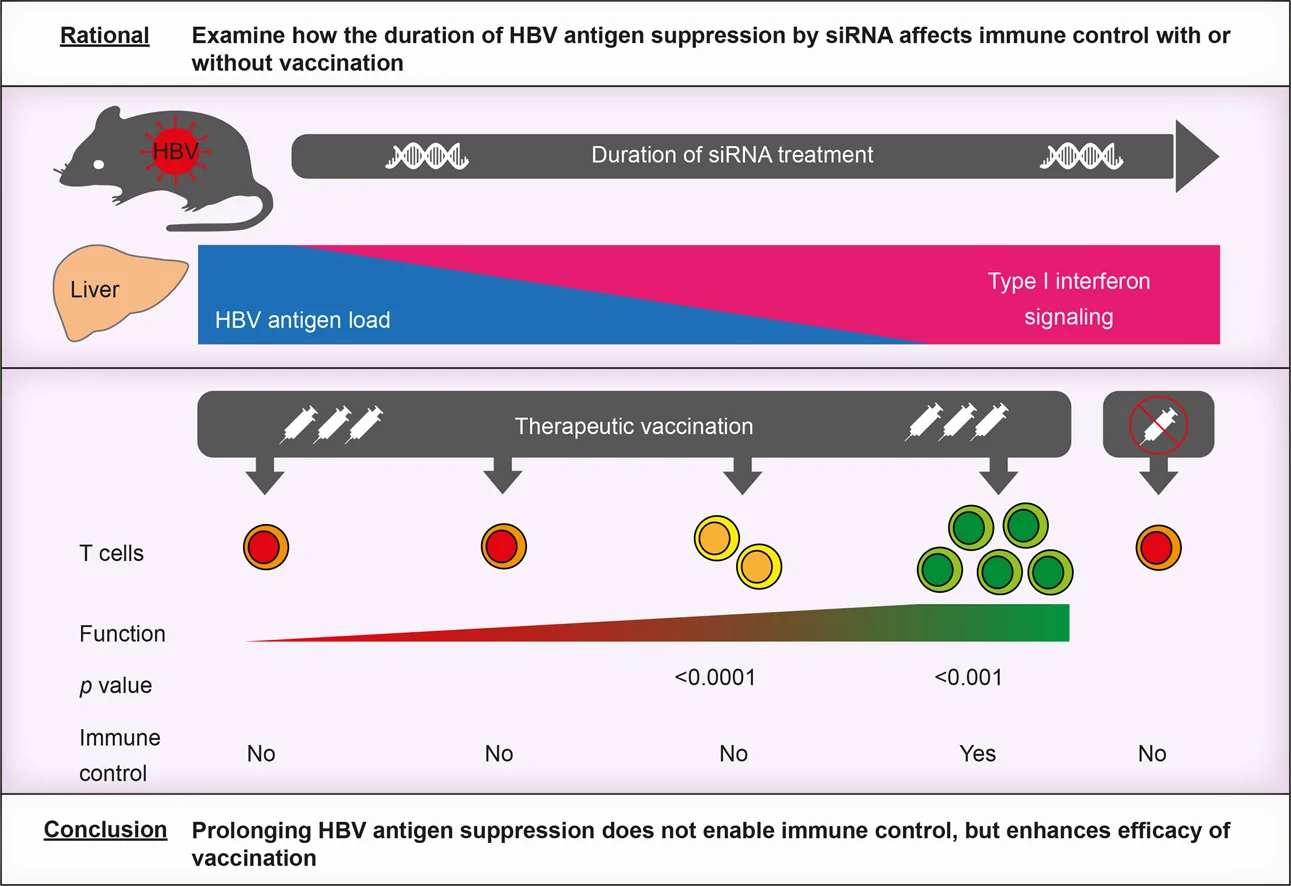

## Introduction

HBV infection is a major cause of human suffering and death. The World Health Organization (WHO) estimates that in 2022, around 254 million people were chronically infected with HBV, and that 1.1 million died of the consequences.^1^ HBV causes more casualties than malaria or HIV, almost reaching the number of deaths caused by tuberculosis.^2^ Without appropriate intervention, prognoses state that HBV may even kill more people than these three pathogens combined by 2040.^3^

One reason why HBV is causing such tremendous harm is that a curative therapy is lacking. The current standard of care, nucleoside analogs (NUCs), suppress the release of HBV but do not target HBV persistence or antigen expression that induces immunotolerance and has carcinogenic properties.^4^^,^^5^ HBV persists as a minichromosome in the nucleus of infected cells, the so-called covalently closed circular DNA (cccDNA), which drives HBV replication and antigen expression.^6^ However, integrated HBV-DNA can also contribute to antigen expression.^7^

We previously presented an approach in which we first suppressed immunoinhibitory viral antigens using an siRNA and 2 months later immunized the animals with a therapeutic hepatitis B vaccine (*TherVacB*). *TherVacB* exploits a heterologous prime-boost strategy to optimally stimulate broad antiviral immunity, particularly HBV-specific T-cell responses. Combining small interfering RNA (siRNA) and *TherVacB* induced robust antiviral immunity, achieving HBV control in HBV-carrier mice.^8^ In contrast, neither restricting HBV replication with NUCs^8^ nor depleting HBeAg^9^ or HBsAg^10^ from the blood of animals allowed *TherVacB* to overcome HBV-specific immunotolerance.

Several siRNAs and antisense oligonucleotides (ASOs) have demonstrated safety and the potential to suppress HBV antigens in patients with chronic hepatitis B.[11], [12], [13], [14] Combinations of siRNAs with immunostimulatory agents such as an fragment crystallizable-optimized and T-cell-stimulating^15^ monoclonal anti-HBs antibody,^16^ pegylated interferon (IFN) alpha-2a,^17^ a TLR8 agonist, a PD-1 antagonist, or therapeutic vaccines are clinically evaluated.^18^

The clinical success of such combinatorial treatment regimens may depend on the baseline HBsAg level, the optimal siRNA dose, and the duration of antigen suppression before immune stimulation. The most important question remaining, however, is whether long-term antigen reduction can lead to spontaneous recovery of HBV immune control, and immunotherapy may be omitted. In this study, we addressed these questions in preclinical HBV-carrier mouse models.

## Materials and methods

### Animal experimentation

Animal experiments were approved by the Animal Care and Use Committee of Upper Bavaria (permission number: 55.2-1-54-2532-202-12) and performed in accordance with the regulations of the German Society for Laboratory Animal Science (GV-SOLAS) and the 3R principles. Mice were housed in a specific-pathogen-free (SPF) facility at the proper biosafety level in accordance with institutional protocols. HBVxfs-transgenic mice carrying a 1.3-fold overlength HBV genome (genotype D, serotype ayw) on a C57BL/6J background [haplotype H-2^b/b^]^19^ were treated at age 8–12 weeks. Alternatively, HBV-carrier mice were established by i.v. injection of an adeno-associated virus (AAV) encoding a 1.2-fold overlength HBV genome (AAV-HBV) of genotype D into 8-week-old male C57BL/6J mice.^8^ Animals were allocated to age-matched groups based on similar HBsAg and HBeAg levels.

### HBV-specific RNAi therapy

HBV-specific siRNAs (siHBV1, siHBV2) were chemically modified to increase their stability^20^ and conjugated to N-acetylgalactosamine (GalNAc) to improve liver uptake.^21^ AAV serotype 8 vectors expressed shRNA targeting the common 3′-end of all HBV transcripts (AAV-shHBV).^8^^,^^22^ Control siRNA (siCtrl) and AAV expressing control shRNA (AAV-shCtrl) targeted human α_1_-antitrypsin.^22^

### Therapeutic hepatitis B vaccine

Briefly, mice were immunized on Days 0 and 14 twice with 15 μg of HBsAg i.m. (genotype A, adw) expressed in yeast and 15 μg of HBV core antigen (genotype D, ayw) particles purified from *Escherichia coli* (kindly provided by APP Latvijas Biomedicinas, Riga, Latvia), forming virus-like particles adjuvanted with 10 μg cyclic di-adenylate monophosphate (c-di-AMP) (InvivoGen, San Diego, CA, USA).^8^^,^^23^ On Day 28, mice were boosted by i.m. injection of 5×10^7^ plaque-forming units each of recombinant Modified Vaccinia Ankara virus (MVA) vectors expressing HBV S or HBV core protein (both genotype D, ayw).

### Serological analyses

HBsAg, HBeAg, and anti-HBs levels were quantified on an Architect^TM^ platform after serum dilution (HBsAg: Ref6C36-44, cut-off: 0.25 IU/ml; HBeAg: Ref.6C32-27 with Quantitative Calibrators (Ref.7P24-01), cut-off: 0.20 PEI U/ml; anti-HBs: Ref.7C18-2, cut-off: 12.5 mIU/ml) (Abbott Laboratories, Wiesbaden, Germany). Anti-HBe was determined using the Enzygnost^TM^ anti-HBe monoclonal test on the BEPIII platform (Siemens Healthcare, Eschborn, Germany). Serum alanine aminotransferase (ALT) activity was measured in a 1:4 dilution using the Reflotron® GPT/ALT test (Roche Diagnostics, Mannheim, Germany). Values and cut-offs of quantitative tests are given after correction for the respective dilution.

### Analysis of HBV-DNA and RNA

DNA was extracted from 25 μl of serum using the QIAamp MinElute Virus Spin Kit (Qiagen, Hilden, Germany) and eluted into 50 μl H_2_O. Quantitative HBV-specific real-time PCR (lower limit of quantification [LLOQ]: 1.705 copies/μl serum) was performed on an Applied Biosystems® 7500 Real-time PCR system (Thermo Fisher Scientific, Darmstadt, Germany) using primers HBV1464_fw/HBV1599_rev and a Taqman probe. RNA was extracted from livers using the RNeasy Mini kit (Qiagen) and reverse transcribed into cDNA using the SuperScript III kit (Thermo Fisher Scientific). HBV transcripts were amplified by real-time RT-PCR on a LightCycler® 480 Instrument II (Roche Diagnostics) with primers to detect only HBV 3.5-kb transcripts that mainly consist of HBV pregenomic RNA. Results were normalized to glyceraldehyde 3-phosphate dehydrogenase (GAPDH) expression. For primer/probe sequences and cycling conditions, see Table S1.

### Intracellular cytokine staining

T cells were stimulated overnight in the presence of 1 mg/ml brefeldin A (Sigma-Aldrich, Taufkirchen, Germany) with 1 μg/ml of peptides MVA_B8R_, OVA_S8L,_ or HBV S_190_, C_93,_ or S_208-215_ (S_208_; IVSPFIPL) or peptide pools covering HBV core (genotype D, aa 70-157) or S (genotype D, aa 145-226). Cell-surface staining was performed using anti-CD8 (clone 56.6-7) and anti-CD4 (cluster of differentiation 4) antibodies (clone L3T4; BD Biosciences, Heidelberg, Germany) after exclusion of dead cells. Staining was performed as described^23^ using an anti-IFNγ antibody (clone XMG1.2; eBioscience) and an anti-tumor necrosis factor (TNF) antibody (clone MP6-XT22; BD Biosciences). Data were acquired on a CytoflexS flow cytometer (Beckmann Coulter) and analyzed using FlowJo software (Tree Star, Ashland, OR, USA). Data are presented as relative values after background subtraction determined using the OVA_S8L_ peptide.

Additional methods are available in the supplementary data.

## Results

### Suppression of HBV antigens reaches a threshold irrespective of the siRNA dose

**Fig. 1:**
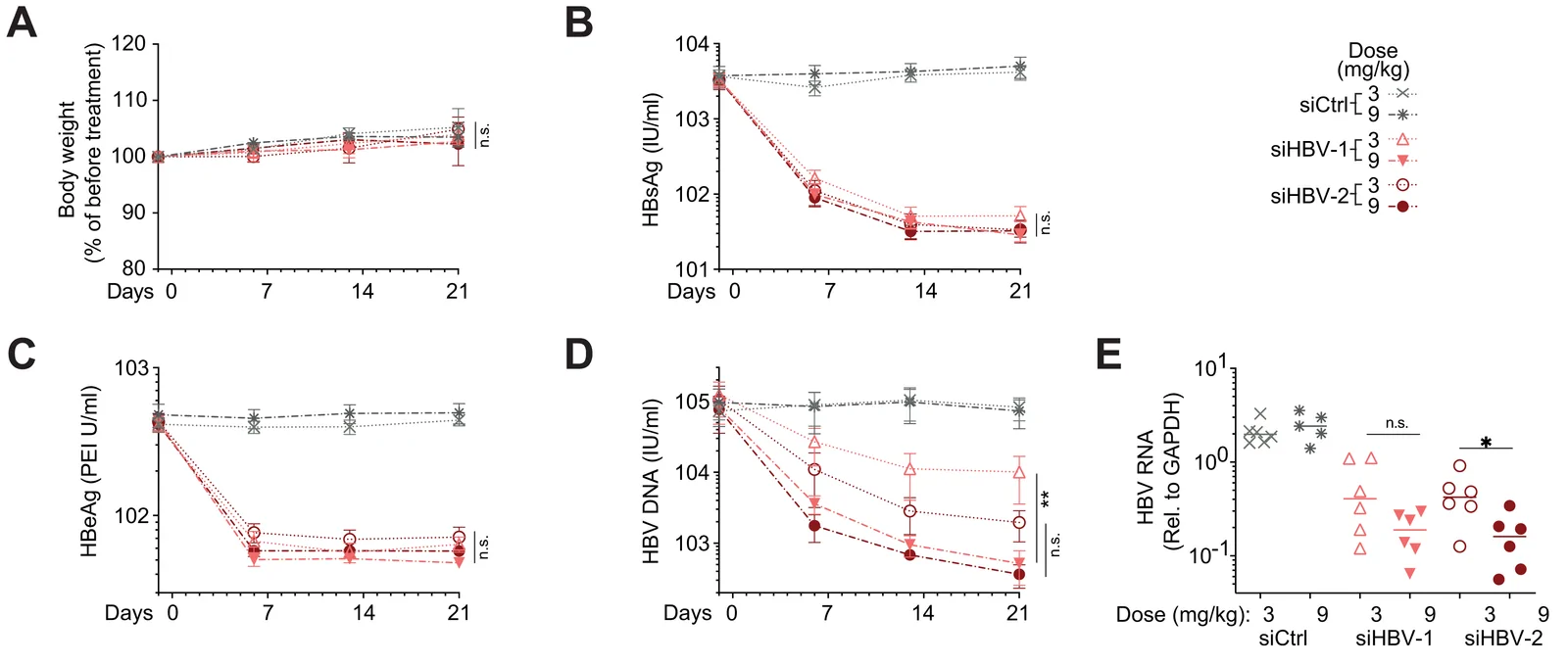
**Influence of siRNA dosing on suppression of viral parameters.** HBV-transgenic mice (HBVxfs line) were treated subcutaneously with either 3 or 9 mg/kg body weight of chemically modified and N-acetylgalactosamine (GalNAc)-coupled siRNAs that target a common region of HBV transcripts (siHBV-1 and siHBV-2) or a control siRNA (siCtrl). (A) The body weight of animals was measured at the indicated time points. Serum levels of (B) HBsAg and (C) HBeAg were determined using chemiluminescence immunoassays, and (D) HBV-DNA by quantitative PCR. (E) Intrahepatic HBV-RNA levels were determined using reverse-transcriptase PCR from liver lysates and presented relative to glyceraldehyde 3-phosphate dehydrogenase (GAPDH) levels. The 9 mg/kg siCtrl group contained five animals and the other experimental groups six animals. In panels A–D, mean and standard error of the mean (SEM), in panel E, median values are given. Statistical differences were calculated using an unpaired *t* test. ns, non-significant; **∗***p* <0.05; **∗∗***p* <0.01. siRNA, small Interfering RNA.

To suppress HBV antigen expression in the liver, we used two highly potent HBV-specific siRNAs that bind a common region of the HBV transcripts and suppress all viral antigens.^8^ They were coupled to GalNAc to allow efficient hepatocyte targeting.^24^ We first investigated whether increasing the siRNA dose, compared with previous studies,^8^^,^^10^ would enhance antigen suppression. We subcutaneously injected HBV-transgenic mice expressing high HBV antigen levels (HBVxfs line;^25^ HBsAg levels: 2.000–3.000 IU/ml; HBeAg: 30–60 PEI U/ml; Fig. 1A,B) with either 3 or 9 mg/kg body weight siHBV-1 or siHBV-2. An siRNA targeting human alpha-1 antitrypsin served as control (siCtrl). Both doses were well tolerated in short-term treatment, and animals did not lose weight (Fig. 1A).

The suppression of HBsAg and HBeAg levels was comparable in all animals irrespective of whether they received 3 or 9 mg/kg of siHBV-1 or siHBV-2 (Fig. 1B,C). The higher siRNA dose led to greater knockdown of parameters associated with HBV replication, including serum HBV-DNA (Fig. 1D) and intrahepatic HBV-RNA levels (Fig. 1E). However, this effect is not clinically relevant, as HBV replication can already be efficiently blocked using NUCs, and was considered a minor advantage. Therefore, we selected the 3 mg/kg dose for further experiments.

### Prolonging RNAi pretreatment enhances the efficacy of therapeutic vaccination

**Fig. 2:**
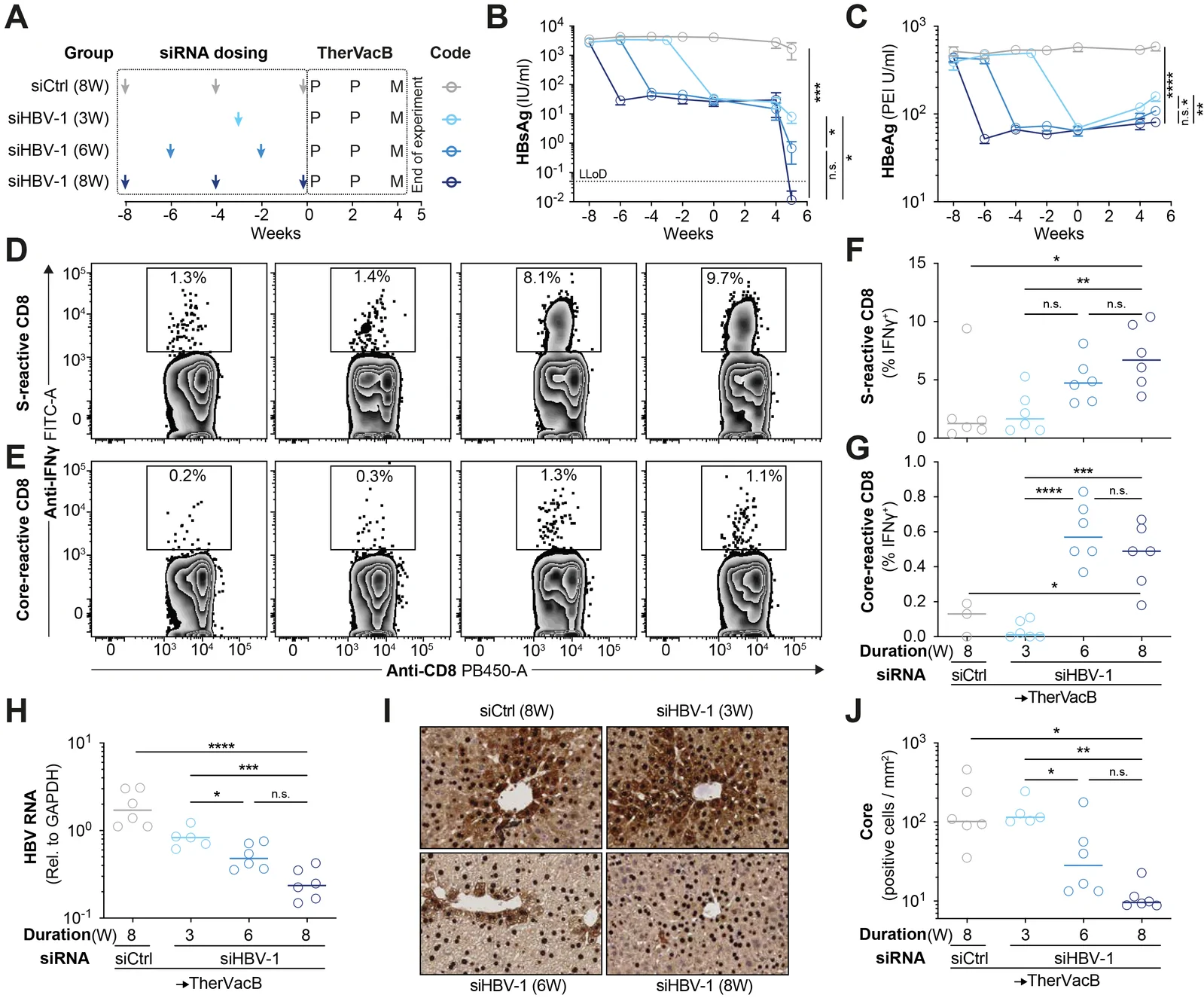
**Influence of the duration of siRNA pretreatment on the efficacy of therapeutic vaccination.** HBV-transgenic mice (HBVxfs line) were treated subcutaneously with 3 mg/kg body weight of an HBV-specific siRNA (siHBV) starting at different time points before vaccination. (A) Scheme depicting time points of siRNA applications and immunizations. Therapeutic vaccination consisted of two protein primes (P) and a boost with a Modified Vaccinia Ankara virus vector (M). (B,C) Serum levels of (B) HBsAg and (C) HBeAg were determined using chemiluminescence immunoassays. (D–G) Intrahepatic HBV-specific CD8 T-cell responses were determined by intracellular cytokine staining for interferon-gamma (IFNγ) following *ex vivo* re-stimulation with HBV-derived peptides specific for (D,F) HBs (S_208_) or (E,G) HBcore (C_93_). Panels D and E show representative flow cytometry plots and F and G quantification of reactive CD8 T cells. (H) Intrahepatic HBV-RNA levels were determined using reverse-transcriptase PCR from liver lysates and presented relative to glyceraldehyde 3-phosphate dehydrogenase (GAPDH) levels. (I*,*J) Histochemical staining of liver sections for HBc. (I) Representative images and (J) quantification of HBc-expressing hepatocytes. Each experimental group contained six animals. In panels B and C, the mean and standard error, in panels F–H,J, the median is indicated. Statistical differences were calculated using one-way ANOVA with multiple comparison corrections. ns, non-significant; **∗***p* <0.05; **∗∗***p* <0.01; **∗∗∗***p* <0.001; **∗∗∗∗***p* <0.0001. LLoD, lower limit of detection; siCtrl, control small interfering RNA (non-targeting control); w, week.

In the next step, we investigated whether the duration of HBV antigen suppression with siRNA affected the efficacy of subsequent therapeutic vaccination using *TherVacB*. For this, we initiated siRNA treatment in HBV-transgenic mice (HBVxfs-tg line) for 3, 6, and 8 weeks before vaccination. Because a repeat injection of GalNAc-coupled siRNA is required every 4 weeks,^14^ the latter two groups received repeated injections while the first group received only one (Fig. 2A). Following s.c. injections of siHBV-1, serum levels of HBsAg dropped within 2 weeks by 2 log_10_ and HBeAg levels by 1 log_10_ (Fig. 2B,C) and remained constant thereafter. Before applying *TherVacB*, all siRNA-treated mice had comparable, low serum antigen levels independent of the duration of siRNA pretreatment.

After therapeutic vaccination, HBsAg levels decreased further. Interestingly, HBsAg levels dropped more significantly in mice that had received several siRNA doses, over a longer period (Fig. 2B). The longer siRNA pretreatment, during which the animals received two siRNA doses, allowed the therapeutic vaccine to induce stronger intrahepatic cluster of differentiation 8 (CD8) T-cell responses (Fig. 2D–G) as observed via flow cytometry (see Fig. S1 for gating strategy) following *ex vivo* stimulation with HBV-specific peptides. The stronger HBV-specific CD8 T-cell response in mice that had received a longer siRNA pretreatment enabled an enhanced suppression of intrahepatic HBV replication as illustrated by decreased HBV-RNA levels (Fig. 2H) and reduced numbers of remaining HBcore^+^ hepatocytes (Fig. 2I,J). Thus, mice that had received repeated siHBV-1 doses over a longer period before vaccination presented a stronger antiviral response.

To determine whether the additional siRNA doses that the mice in the 6-week and 8-week groups had received or differences in intrahepatic HBV antigen levels not reflected in serum were responsible for the differences observed, we repeated the experiment, but this time used shRNA-expressing AAV vectors to achieve a constant expression of shRNA to trigger RNA-interference (RNAi). This experimental set-up confirmed that prolonging antigen knockdown from 3 to 6 weeks increased *TherVacB’s* ability to induce antiviral T-cell responses (Fig. 2). Taken together, these experiments indicated that mice responded better to *TherVacB* after prolonged suppression of HBV antigen expression in the liver.

### Intrahepatic antigen levels drop more slowly than serum levels and correlate with the efficacy of therapeutic vaccination

To understand why prolonged suppression of antigen expression is beneficial for the induction of antiviral immunity, although suppression of serum HBsAg and HBeAg was already achieved after 2 weeks, we treated HBV-transgenic mice using AAV-shRNA vectors. The HBV-tg mouse model ensured constant expression of HBV antigens, and the AAV-shRNA allowed for a constant suppression of HBV antigen expression in hepatocytes. We terminated the experiment after 3, 5.5, and 8 weeks to match the time points at which we started vaccination in the earlier experiments.

**Fig. 3:**
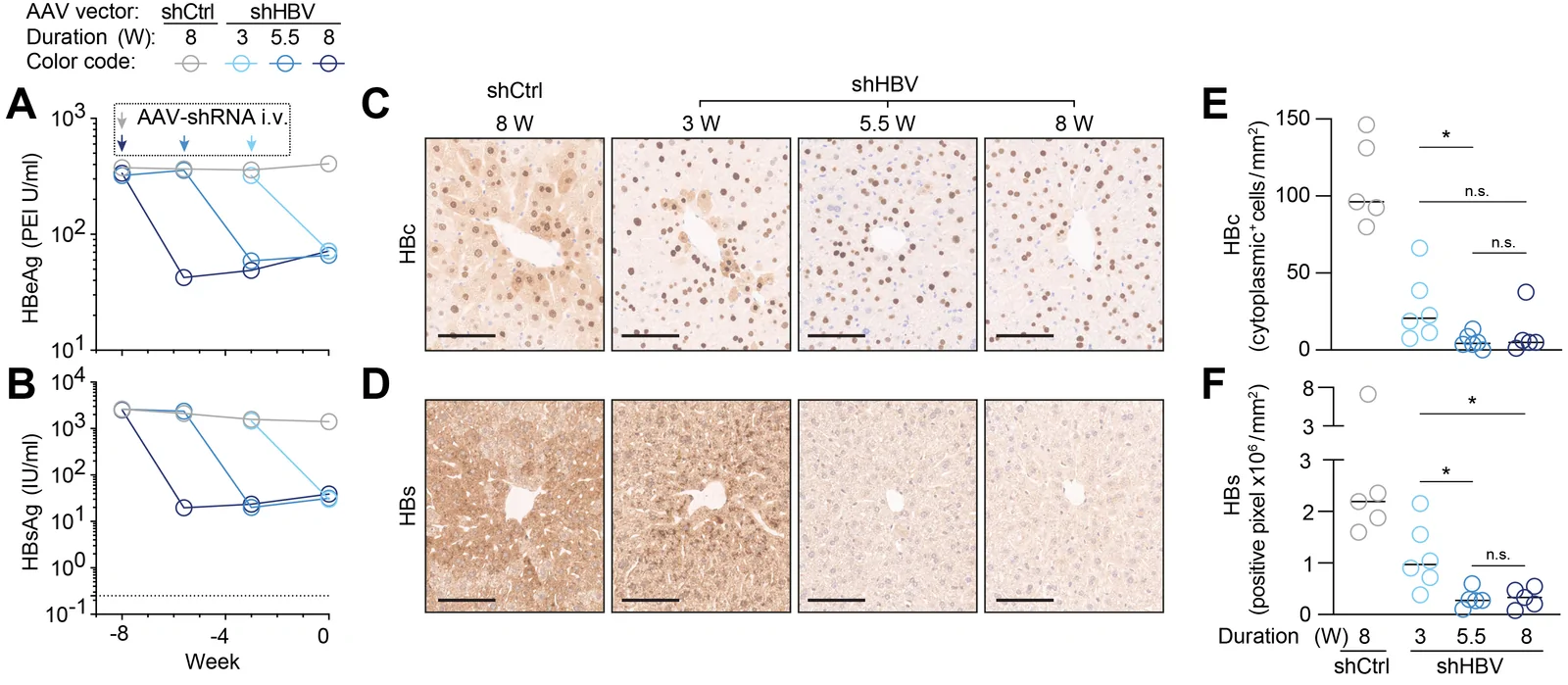
**Dynamics of intrahepatic HBV antigen expression following RNAi treatment.** HBV-transgenic mice (HBVxfs line) were treated at varying time points intravenously with 10^11^ genome equivalents of an AAV vector expressing an HBV-specific (shHBV) or a control shRNA (shCtrl). Serum levels of HBsAg (A) and HBeAg (B) were determined using chemiluminescence immunoassays. Intrahepatic expression levels of (C) HBs and (D) HBc were analyzed by immunohistochemical staining following quantification of (D) HBc-positive cells or (E) area staining HBs positive. Experimental groups contained five (shCtrl, shHBV 5.5W) or six (shHBV 3W and shHBV 8W) animals. Bars at the bottom left of each image indicate 100 μm. Graphs in A and B show mean and bars in D and E represent the median. Statistical differences were calculated using one-way ANOVA with multiple comparison correction. ns, non-significant; **∗***p* <0.05. w, week.

HBcAg and HBsAg staining of the livers enabled analysis of the dynamics of intrahepatic antigen expression. As expected, RNAi treatment reduced viral antigen levels not only in serum (Fig. 3A,B), but also antigen expression in the liver (Fig. 3C–F). The observed dynamics, however, were slower than the reductions in HBsAg and HBeAg levels in the animals’ blood. Although serum HBsAg and HBeAg reached their maximum suppression already within 2–3 weeks (Fig. 3A,B), HBsAg and HBcore suppression in the livers was still incomplete after 3 weeks and reached minimal levels only after 5.5 weeks of RNAi (Fig. 3E,F).

To investigate whether intrahepatic immune cells contribute to this effect, we studied the proportional distribution of potentially immunosuppressive cells in the liver during RNAi. Myeloid-derived suppressor cells (MDSCs),^26^ Kupffer cells,^27^ natural killer (NK) cells,^28^ and regulatory T cells (Tregs)^29^ have been reported to inhibit HBV-specific immune responses. We isolated liver-associated lymphocytes from treated mice and quantified cell populations after staining characteristic surface markers by flow cytometry. Overall, the duration of RNAi treatment did not significantly affect the composition of intrahepatic immune cells. Neither the number of Ly6C^+^ nor Ly6G^+^ Myeloid cells, Kupffer cells, NK cells, NK T cells, nor regulatory T cells significantly differed when RNAi was maintained for a longer time period (Fig. S3).

### Restoration of the type I IFN response correlates with the duration of HBV antigen suppression

**Fig. 4:**
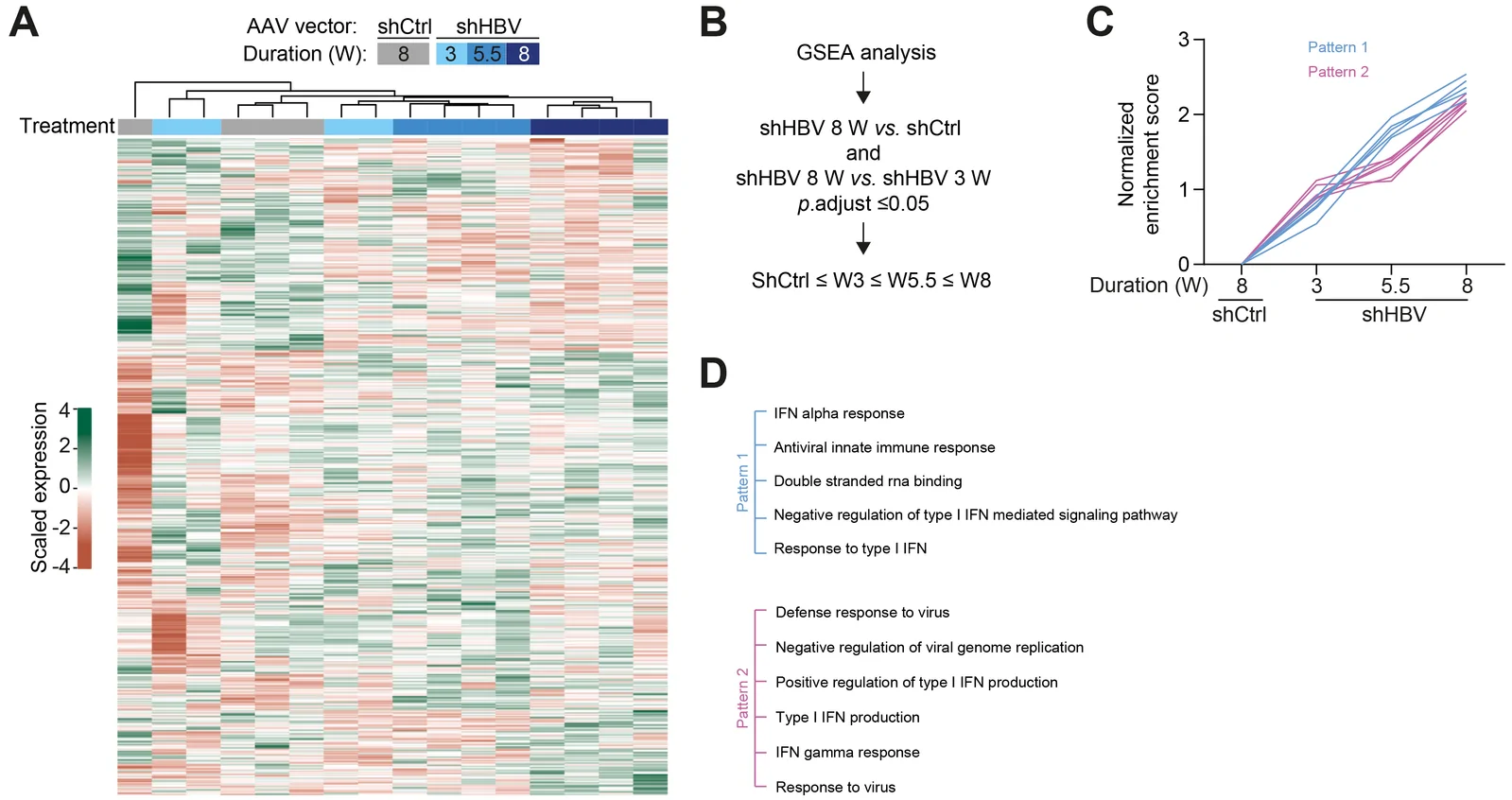
**Influence of the duration of RNAi treatment on intrahepatic signaling pathways.** HBV-transgenic mice (HBVxfs line) were treated intravenously at varying time points (3, 5.5, or 8 weeks before the end of the experiment) with 10^11^ genome equivalents of an adeno-associated virus (AAV) vector that expresses an HBV-specific short-hairpin (shHBV) or a control shRNA (shCtrl). RNA was extracted from whole liver lysate of four animals per group and after cDNA synthesis bulk sequenced on the Illumina NovaSeq X Plus platform. (A) Overview over differentially expressed genes (adjusted *p* <0.05; LogFC >1) between treatment groups. (B) Algorithm used to identify signaling pathways of which the enrichment correlated with the observed phenotype. (C) Dynamic of Gene Set Enrichment Analysis (GSEA) of (D) pathways that were identified according to the algorithm shown in (B).

To identify factors enabling successful immunization, we analyzed how the different durations of HBV antigen reduction affected the intrahepatic transcriptome using bulk RNA sequencing of liver lysates. When comparing animals treated with AAV-shHBV *vs*. AAV-shCtrl 8 weeks earlier (Fig. S4), we found that 610 genes were significantly regulated (73 genes if assuming a LogFC >1). In contrast, 1,600 genes (236 genes when assuming LogFC >1) were regulated between animals treated with AAV-shHBV for 8 weeks *vs*. 3 weeks, indicating that major transcriptomic changes occurred during this time frame (Fig. 4A; Table S2).

A Gene Set Enrichment Analysis (GSEA) was performed to identify involved signaling cascades. We selected pathways for which the enrichment correlated with the observed immune phenotype, considering the following (Fig. 4B): (i) A significant enrichment in animals treated with shHBV for 8 weeks compared with shCtrl. (ii) A significantly stronger enrichment in animals treated with shHBV for 8 weeks compared with 3 weeks. (iii) No significant up- or downregulation during the time course.

Eleven pathways fulfilled these criteria and were thus considered to correlate with the observed immune phenotype. Of these, five pathways showed a constant increase in enrichment (pattern 1 in Fig. 4C), and six showed a delayed increase between week 3 and week 5.5 (pattern 2 in Fig. 4C). Interestingly, there was a substantial overlay among the genes involved (Fig. S5), with the dominant process being a restoration of the type I IFN response. Other pathways were related to processes such as double-stranded RNA binding, antiviral innate immune response, a defense response to viruses, or an IFN-gamma response. This suggests that suppression of innate immunity, including type I IFN signaling, contributed to HBV immunotolerance in our model.

### Long-term antiviral siRNA therapy does not achieve HBV cure

**Fig. 5:**
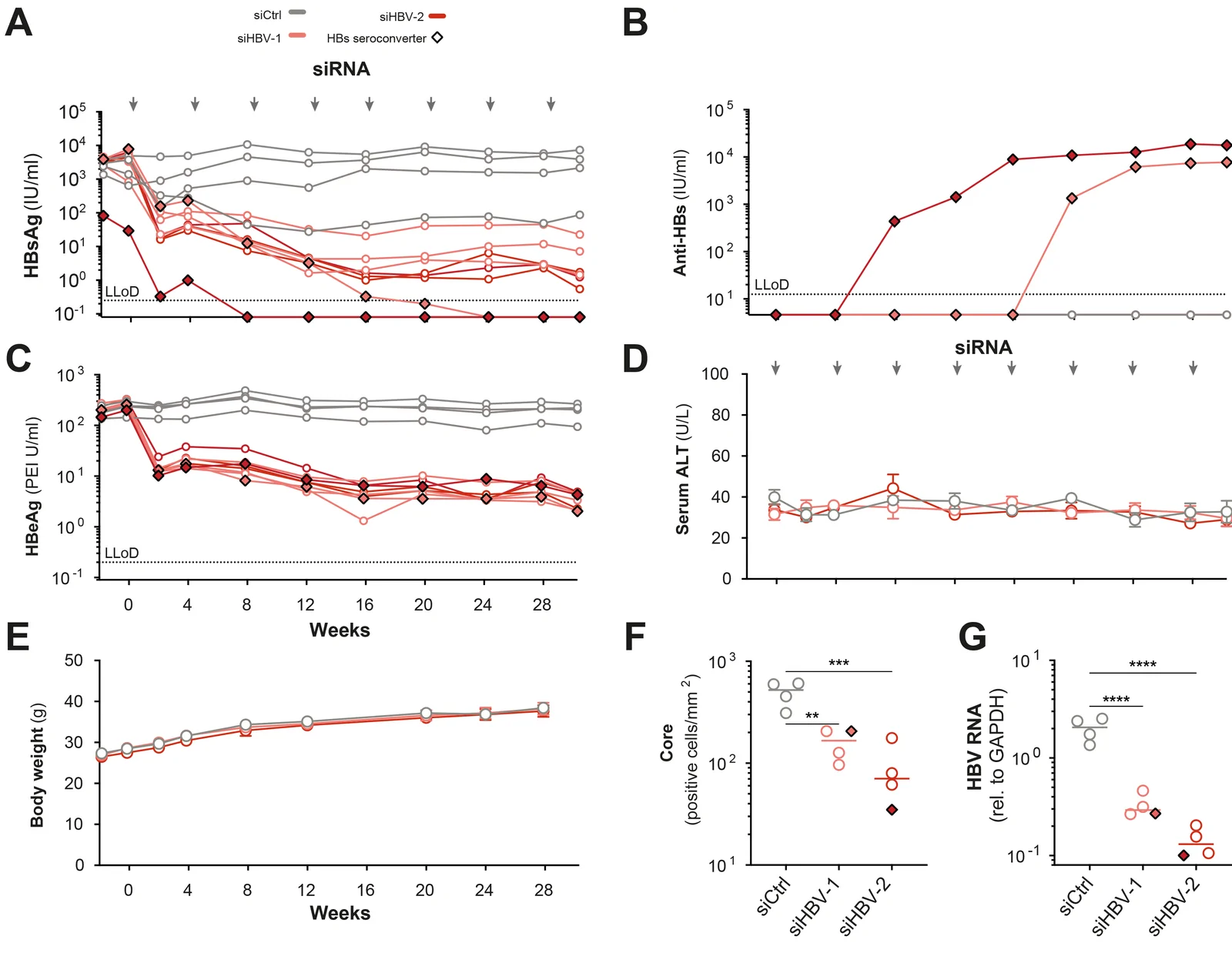
**Effect of long-term treatment with antiviral siRNAs on antigenemia and tolerability.** C57/BL6 mice were intravenously injected with 2×10^10^ geq of AAV-HBV to establish an HBV-carrier state. After 4 weeks, mice received monthly s.c. injections of HBV-specific siRNAs (siHBV-1 [lighter lines] or siHBV-2 [darker lines]) or a control siRNA (siCtrl, gray lines) for 8 months. The course of HBV infection was evaluated by measuring serum levels of (A) HBsAg, (B) anti-HBs antibodies, and (C) HBeAg using chemiluminescence immunoassays. Side effects were monitored by determining (D) activity of alanine aminotransferase (ALT) in serum or (E) measuring body weight of mice. (F) Immunohistochemical staining of mouse livers for HBc. Counts of cytoplasmic HBc-positive cells are indicated. (G) HBV pregenomic RNA was quantified in livers using reverse-transcriptase PCR. Bars indicate median. Panels A–C show values of individual mice, and graphs D and E mean values and SEM. Each experimental group contained four animals. Bars in F and G indicate median. Statistical differences were calculated using one-way ANOVA with multiple comparison correction. **∗∗***p* <0.01; **∗∗∗***p* <0.001; **∗∗∗∗***p* <0.0001; LLoD, lower limit of detection; siRNA, small Interfering RNA.

Encouraged by the finding that increasing the duration of siRNA pretreatment enhanced the efficacy of *TherVacB*, we wondered whether further prolonging siRNA treatment would allow restoration of HBV-specific immunity without the need for additional immune stimulation. In contrast to our previous experiments, we instead opted for the AAV-HBV model of chronic hepatitis B, in which cure can theoretically be achieved. Using 2×10^10^ genome equivalents (geq) AAV-HBV per mouse, we established high-level HBV-carrier mice with serum HBsAg levels between 2,000 and 10,000 IU/ml, except one mouse with HBsAg 100 IU/ml (Fig. 5A), and HBeAg levels of 200 to 400 PEI U/ml (Fig. 5C).

After 4 weeks, mice received 3 μg/g body weight siHBV-1, siHBV-2, or the control siRNA s.c. every 4 weeks over a period of 28 weeks. In contrast to previous experiments, we did not vaccinate the mice. HBV antigen levels dropped sharply following the first siRNA application, with siHBV-2 showing higher antiviral activity than siHBV-1, as observed previously.^8^ Four weeks after the first siRNA application, HBsAg serum levels had dropped by 1.5 to 2.5 log_10_, and HBeAg levels by 1.0 log_10_. HBsAg levels continued to drop by a further 1.5 log_10_, and HBeAg levels by a further 0.5 log_10,_ reaching a plateau at week 16. Two animals, one starting at 10,000 IU/ml HBsAg treated with siHBV-1, and the animal with baseline HBsAg levels of only 100 IU/ml HBsAg treated with siHBV-2, showed a continuous drop of serum HBsAg levels to below the detection limit, accompanied by anti-HBs seroconversion (Fig. 5B). However, their HBeAg levels remained stably positive, indicating persistence of the HBV genome. Even eight siRNA injections did not lead to detectable side effects, with no ALT elevations (Fig. 5D) and no weight loss (Fig. 5E). At the end of treatment, we observed a significant reduction in the number of hepatocytes staining positive for HBV core (Fig. 5F) and hepatic HBV-RNA (Fig. 5G), but no elimination of HBV. This showed that long-term suppression of HBV antigens did not achieve cure or immune control of HBV, even when HBsAg seroconversion was achieved.

### Long-term siRNA monotherapy does not lead to reconstitution of T-cell immunity

**Fig. 6:**
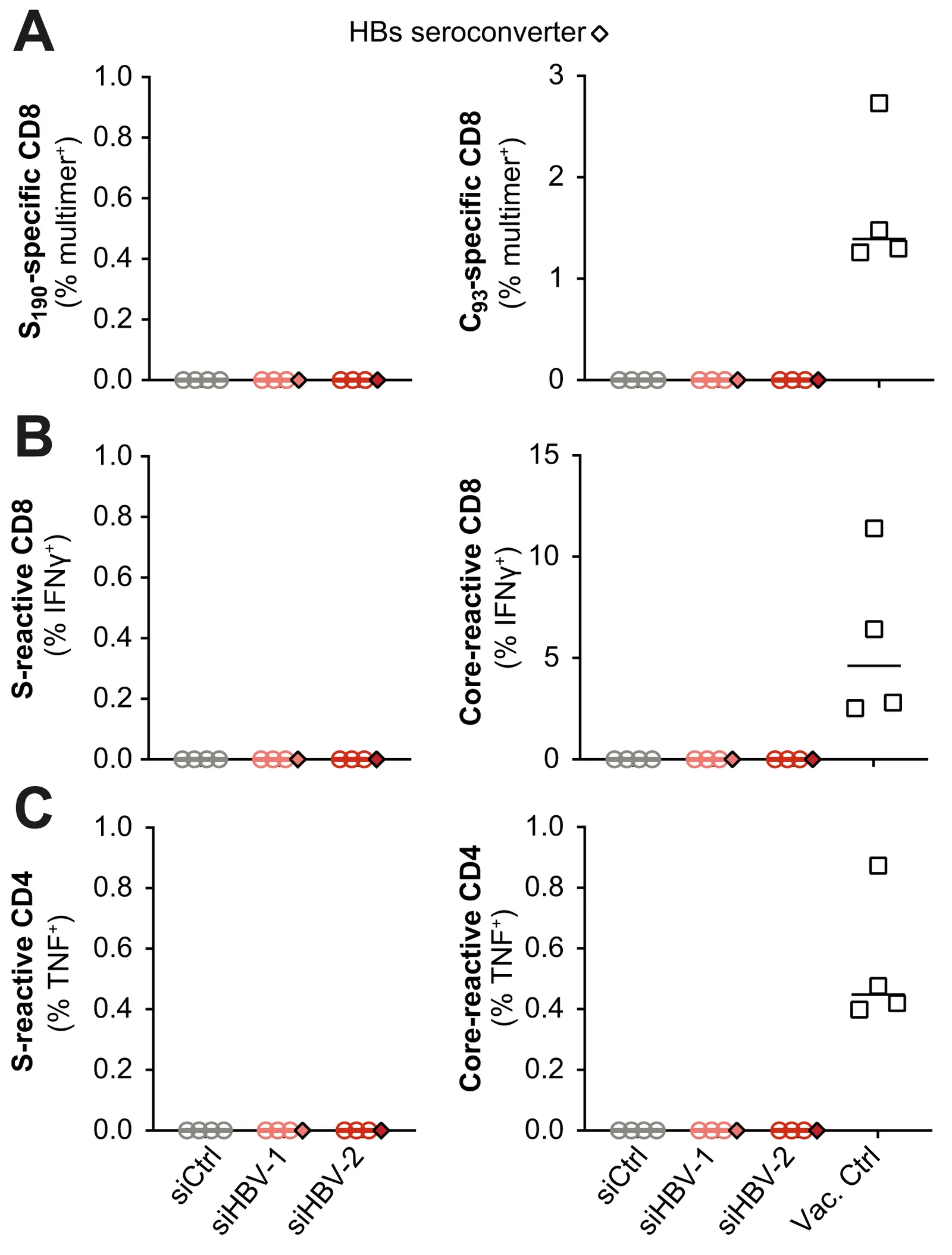
**Effect of long-term treatment with antiviral siRNAs on HBV-specific T-cell responses.** C57/BL6 mice were i.v. injected with 2×10^10^ geq of AAV-HBV to establish an HBV-carrier state. Starting after 4 weeks, mice received monthly s.c. injections HBV-specific siRNAs or a control siRNA (siCtrl) as indicated for 8 months (n = 4). Two weeks after the last siRNA application, liver-associated lymphocytes were isolated and HBV-specific T-cell responses analyzed. A positive control group (Vac. Ctrl) was not infected with AAV-HBV but was immunized with adjuvanted HBcoreAg followed by an HBcore-expressing MVA. (A) Intrahepatic, HBV-specific CD8 T cells were detected using MHC-I multimers specific for the HBsAg-derived S_190_ epitope (left graph) or the HBc-derived C_93_ epitope (right graph). Frequency of HBV-specific T cells is given. HBV-reactive (B) CD8 or (C) CD4 T cells were analyzed by *ex vivo* stimulation with peptide pools covering the entire HBV S- (graphs on left) or core-protein (graphs on right), and subjected to intracellular cytokine staining. (B) IFNy^+^ and (C) TNF^+^ cells were quantified by flow cytometry. Each experimental group contained four animals. Bars indicate median. AAV-HBV, AAV expressing the HBV genome; siRNA, small Interfering RNA. MHC, major histocompatibility complex.

To investigate whether long-term suppression of HBV antigen expression would allow for reconstitution of T-cell immunity in our young, fully immune-competent animals, we isolated immune cells from livers and spleens and assessed antiviral T-cell responses using HBV-specific multimer staining and intracellular cytokine staining after 7 months of siRNA treatment. Four mice vaccinated with HBcoreAg following the *TherVacB* scheme served as positive controls. Even after long-term antigen suppression, we could neither detect HBV-specific CD8 T cells using S_190_ and C_93_ multimers (Fig. 6A), nor restimulate CD8 or CD4 T-cell responses with HBV peptide pools (Fig. 6B,C). Interestingly, we could not even detect HBV-specific T-cell responses in the two mice that seroconverted to anti-HBs (marked by diamonds with black borders in Fig. 6), but readily detected them after therapeutic vaccination. We concluded that even long-term reductions in HBV antigens in the liver and blood by siRNA treatment do not allow spontaneous recovery of T-cell immunity in HBV-carrier mice, even when accompanied by seroconversion to anti-HBs.

### A durable HBV control after cessation of siRNA therapy requires immune stimulation

**Fig. 7:**
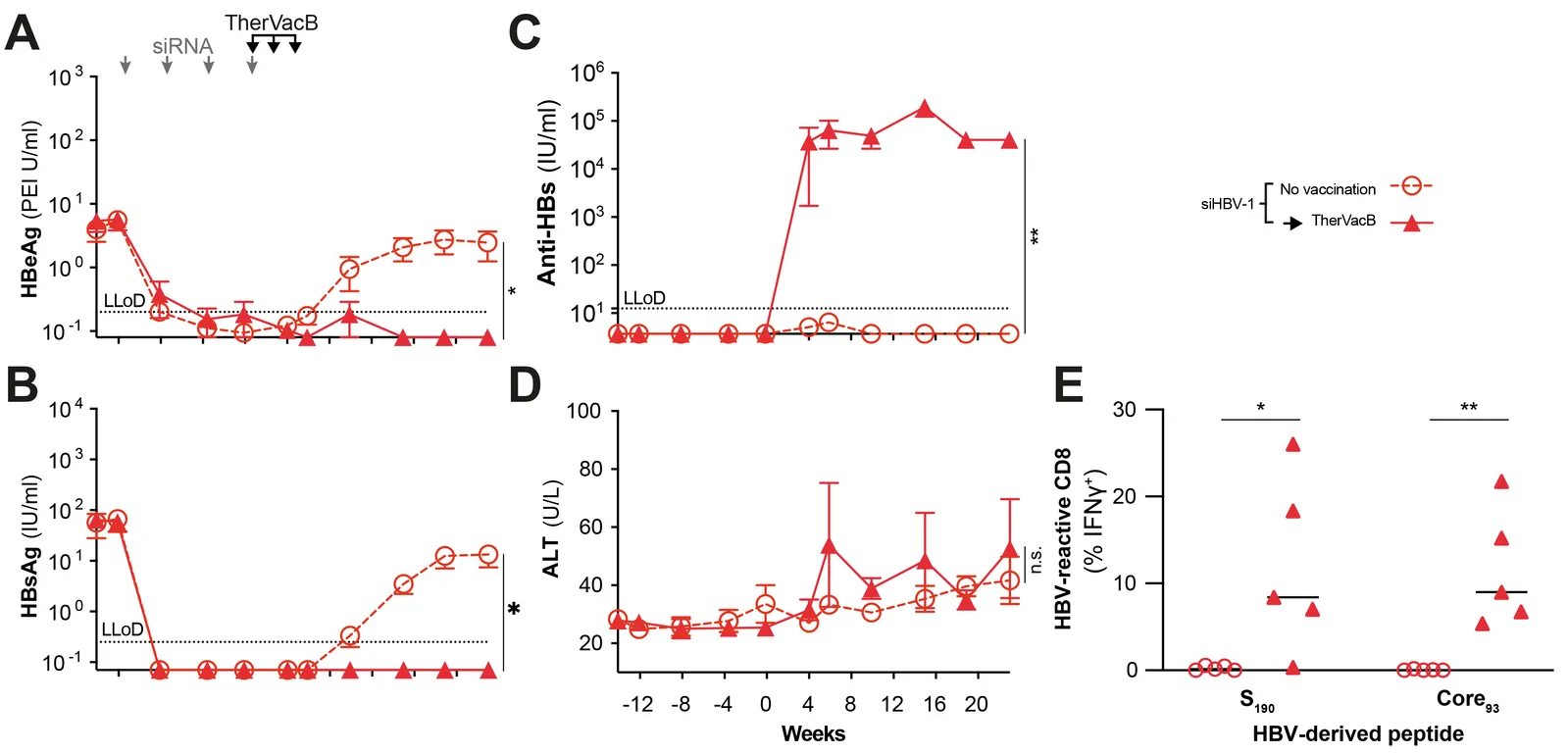
**Effect of HBV antigen suppression to below the detection limit on HBV control and immunity.** C57/BL6 mice were infected with 8×10^8^ geq of AAV-HBV. After 4 weeks, mice were treated with four monthly s.c. injections of 3 mg/kg body weight of an HBV-specific siRNA (siHBV-1). Half of the mice were immunized starting with the last siRNA application, whereas the other mice received no further treatment. HBV control was evaluated by measuring serum levels of (A) HBeAg, (B) HBsAg, (C) anti-HBs antibodies, and (D) ALT activity. (E) Intrahepatic virus-specific CD8 T-cell responses were evaluated 23 weeks after start of TherVacB by *ex vivo* stimulation of liver-associated lymphocytes with immune-dominant S_190_ (left) or C_93_ peptides (right), and the fraction of reactive CD8 T cells determined by flow cytometry after intracellular cytokine staining for IFNγ. Each experimental group contained five mice. Graphs A–D show mean and SEM. Bars in E indicate median. Statistical differences were calculated using an unpaired *t* test. ns, non-significant; **∗***p* <0.05; **∗∗***p* <0.01. IFNγ, interferon-gamma.

Having observed complete loss of serum HBsAg in the two mice that seroconverted to anti-HBs in the previous experiment, we aimed to more systematically investigate whether suppression of viral antigens by siRNA to below the detection limit would enable restoration of HBV-specific immunity without an additional immune stimulus. As one of the two mice that seroconverted to anti-HBs in the previous experiment only had 100 IU/ml HBsAg in the serum at the start of the experiment, and to increase the chance of clearing HBV and restoring antiviral immunity, we decided to repeat the experiment in low-titer HBV-carrier mice. Following injection of 8×10^8^ geq AAV-HBV, mice presented with serum antigen levels of 50–100 IU/ml HBsAg (Fig. 7A) and 5–10 PEI U/ml HBeAg (Fig. 7B). As serum antigen levels continued to drop over four consecutive siRNA doses (Fig. 5A), mice received four siRNA injections, resulting in undetectable levels of serum HBsAg and HBeAg (Fig. 7A,B). After 12 weeks, mice were either vaccinated with *TherVacB* or left untreated to observe whether HBV-specific immunity could control HBV after cessation of siRNA treatment.

In this experiment, none of the non-vaccinated mice seroconverted to anti-HBs. In contrast, all mice that received *TherVacB* developed anti-HBs antibodies (Fig. 7C) and maintained serum HBsAg and HBeAg levels below the detection limit for the remainder of the experiment (Fig. 7A,B). Only vaccinated mice showed slightly elevated ALT levels (*p* = 0.054), indicating T-cell activity (Fig. 7D). At the end of the experiment, HBV-reactive CD8 T cells were readily detected in vaccinated mice (Fig. 7E). The non-vaccinated animals developed no antibody or T-cell responses. Accordingly, they relapsed after stopping siRNA treatment and replicated HBV again, with serum antigen levels returning to baseline (Fig. 7A–E). We concluded that even if starting serum HBV antigen levels are low and then suppressed to below the detection limit by antiviral siRNA for a prolonged period, an immune-stimulatory therapy is necessary to achieve long-lasting HBV control.

## Discussion

In this study, we demonstrated that an siRNA targeting four of five HBV transcripts can significantly lower HBV antigen levels and limit HBV replication. Still, it was not sufficient to restore HBV-specific immunity or prevent a relapse of HBV replication. Restoring antiviral immunity, and in particular HBV-specific T-cell responses, required a combination with *TherVacB*, a therapeutic vaccine designed to activate HBV-specific CD4+ and CD8+ T cells. This held even in mice with a low-level antigen-carrier state (HBsAg <100 IU/ml), which also required additional activation of HBV-specific T-cell immunity for sustained HBV control.

As observed in clinical trials, we did not find a significant benefit in increasing the siRNA dose from 3 to 9 mg/kg body weight.^30^ However, administering four monthly siRNA doses resulted in a sustained reduction in viral antigen levels. When we applied additional siRNA doses, we observed a threshold effect: viral antigens did not decline further. Interestingly, a similar pattern was observed in a phase 2a trial using JNJ-73763989,^31^ in which antigen decline reached a threshold after 32 weeks of siRNA treatment. Biological processes generally occur over longer time frames in humans than in mice, most likely attributable to differences in life spans.^32^ Thus, the observations we made in our mouse models, such as the appearance of a threshold effect after a certain time, the half-life of viral antigens in the liver, or the duration required to suppress viral antigens to break immunotolerance, might translate to humans, but with varying time frames.

During our study, we used two different mouse models of chronic hepatitis B: (i) HBV-transgenic mice (HBVxfs line), which express a consistent level of HBV antigens from birth onward, resulting in high immunotolerance. The limitation of this model, however, is that the integrated HBV cannot be eliminated, and thus, a cure cannot be achieved. (ii) The AAV-HBV model that allows generating HBV-carrier mice at different HBV titers, using a variety of HBV variants^10^ or different mouse strains. After AAV-HBV infection, the HBV template persists episomally, allowing it to be cleared from the liver. This model limits the application of a second AAV vector. We thus used the HBVtg model to continuously express an shRNA using AAV vectors. However, natural HBV infection, during which HBV can spread to uninfected hepatocytes and cause inflammation, is not mimicked by either of the two HBV-carrier models, potentially influencing the dynamics of HBV antigen levels following siRNA therapy. This makes it hard to predict the dynamics of intrahepatic antigen levels following siRNA therapy in the clinical setting.

Another important observation during our study was that the duration of siRNA treatment affected the efficacy of subsequent therapeutic vaccination. Here, mice in which HBV antigens were suppressed for 3 weeks showed a weaker response to therapeutic vaccination than mice in which antigen levels were suppressed for 6 or 8 weeks. In this setting, serum antigen levels did not predict the response of mice to therapeutic vaccination, at least during the initial phase of siRNA treatment. A possible explanation is that HBV antigens in the liver have a longer half-life than those in serum. This finding is consistent with our previous finding that intrahepatic HBV antigen levels are the main predictor of response to vaccination.^10^ Thus, it appears plausible that the increased efficacy of vaccination after 8 weeks compared with 3 weeks of RNAi pretreatment was a result of differences in intrahepatic antigen levels. Therefore, clinical studies assessing the efficacy of immunotherapies should aim to determine initial intrahepatic antigen levels and immune responses using fine-needle biopsies to predict the strength of immune tolerance.^33^

We observed that the duration of HBV antigen suppression inversely correlated with the strength of HBV-specific immunotolerance. Interestingly, in patients with chronic hepatitis B, the duration of HBV persistence inversely correlated with antiviral T-cell responses, with HBsAg-specific T cells decreasing with longer HBsAg exposure.^34^ While we did not observe any significant changes in the frequencies of regulatory immune cell populations in the liver, we identified signaling pathways that became increasingly enriched in liver tissue with longer durations of RNAi pretreatment using bulk RNA sequencing. A dominant pattern following HBV antigen suppression was the activation of innate immune signaling, especially type I IFN signaling. While HBV has previously been labeled as a ‘stealth virus’, as few transcriptomic changes were observed in the livers of HBV-infected chimpanzees,^35^ a more complex picture emerged during recent years. Several studies found that HBV can be recognized by intracellular pattern recognition receptors, including Toll-like receptors (TLRs) 2, 3, and 4, RIG-I-like receptors, and cGAS, leading to innate immune activation in hepatocytes and non-parenchymal liver cells.^36^ Explaining the initial ‘stealth virus’ concept, numerous studies showed that HBV actively suppresses the induction of innate immune responses, especially type I IFN responses.[37], [38], [39], [40], [41], [42], [43], [44], [45], [46] Such type I IFN responses, however, are crucial for clonal expansion and memory formation of antiviral CD8 T cells.^47^ Missing hepatic IFN type I contributes to ineffective and exhausted CD8 T-cell responses.^48^ Moreover, the blockade of INF signaling has been identified as a key mechanism underlying the persistence of lymphatic choriomeningitis virus in the liver.^49^ Thus, the correlation between immune reconstitution and changes in intrahepatic signaling pathways in our study suggests that skewing type IFN signaling is an important mechanism by which HBV establishes immunotolerance.

A crucial finding of our study was that siRNA treatment alone was unable to restore T-cell immunity and achieve a sustained HBV control. This held even if siRNA treatment was applied for up to 7 months, starting HBsAg levels were <100 IU/ml, serum HBsAg and HBeAg levels were suppressed to below the detection limit, and despite anti-HBs seroconversion. This reflects observations from clinical trials, in which siRNA therapy rarely produced sustained effects.^50^ In all scenarios investigated, immune stimulation by therapeutic vaccination was necessary to induce T-cell responses and control HBV after cessation of siRNA treatment, providing important information urgently needed by the field.^50^

Our study also confirms that anti-HBs seroconversion by itself is a poor predictor of lasting HBV control,^51^ and rather reflects the balance between HBsAg and anti-HBs production. HBV control was achieved only in mice that developed HBV-specific T-cell responses. HBV control was characterized by a loss of viral antigen and seroconversion to anti-HBs and anti-HBe. In contrast, AAV-HBV levels in the liver, similar to those of HBV cccDNA, remained stable. Moderately elevated ALT levels indicated that at least some hepatocytes were killed, but persisting AAV-HBV genomes indicated incomplete clearance of HBV-positive cells. Given that most patients who undergo a spontaneous cure also remain cccDNA positive,^52^ this finding reflects the situation observed in patients. However, the single-stranded AAV vectors used to deliver the HBV genome must undergo second-strand synthesis before becoming transcriptionally active, a process known to be ineffective.^53^ Thus, we cannot exclude that at least a fraction of AAV genomes derive from dysfunctional AAV templates that do not express HBV.

In summary, our study answered some critical questions that will help fine-tune combinatorial therapies for chronic hepatitis B. It consolidates the notion that intrahepatic, rather than circulating HBV antigen levels, are the main determinant of HBV immunotolerance, and that T cells are the crucial mediators of HBV control. Finally, it argues for combining antivirals that suppress viral antigen expression in the liver for prolonged time periods before applying an immunotherapy that stimulates antiviral T cells. As a contributing factor, it identified a restoration of type I IFN signaling. If combination treatment regimens are developed to their full potential and treatment is guided by the right biomarkers, the hope of achieving HBV control or even a cure will come into sight.

## Abbreviations

AAV, adeno-associated virus; AAV-HBV, AAV expressing the HBV genome; AAV-shCtrl, AAV expressing control shRNA; ALT, alanine aminotransferase; ASO, antisense oligonucleotide; cccDNA, covalently closed circular DNA; c-di-AMP, cyclic di-adenylate monophosphate; GalNAc, N-acetylgalactosamine; GAPDH, glyceraldehyde 3-phosphate dehydrogenase; geq, genome equivalents; GSEA, Gene Set Enrichment Analysis; IFN, interferon; LLOQ, lower limit of quantification; MDSCs, myeloid-derived suppressor cells; MHC, major histocompatibility complex; MVA, Modified Vaccinia Ankara vector; NK, natural killer cells; NUCs, nucleoside analogs; PEI U/ml, Paul-Ehrlich Institute Units per milliliter; RNAi, RNA-interference; shRNA, short-hairpin RNA; siCtrl, control small interfering RNA (non-targeting control); siRNA, small interfering RNA; SPF, specific-pathogen-free; *TherVacB*, therapeutic vaccine for chronic Hepatitis B; TLR, Toll-like receptor; TNF, tumor necrosis factor; Tregs, regulatory T cells; WHO, World Health Organization.

## Authors’ contributions

Conceived the experiments: TM, UP. Supervised the work and provided grant funding: UP. Performed the experiments and collected data: TM, PH, AK, TB, JS. Analyzed and visualized the data: TM. Provided reagents: DB. Wrote the manuscript: TM, UP. Reviewed the final version: all authors.

## Data availability

The sequencing data is accessible via the NCBI's Gene Expression Omnibus and accession number GSE324334. Further data are available from the corresponding author upon reasonable request.

## Financial support

The study was supported by 10.13039/501100006338VIR Biotechnology, San Francisco, CA, USA, a licensee of the HBV-specific siRNA technology, and the 10.13039/501100001659Deutsche Forschungsgemeinschaft (10.13039/501100001659German Research Foundation), via the Collaborative Research Centers TRR179 (project-ID 272983813, to 10.13039/501100004238UP), TRR338 (project-ID 452881907, to 10.13039/100015870DB and 10.13039/501100004238UP), and RTG2668 (project-ID 435874434, to 10.13039/501100004238UP and 10.13039/100015870DB). The BMFTR supported 10.13039/501100004238UP in the framework of the Cluster4Future program (Cluster for Nucleic Acid Therapeutics Munich, CNATM).

## Conflicts of interest

UP and ADK are named as inventors on a patent application describing the therapeutic vaccination scheme of TherVacB (PCT/EP2017/050553). UP and TM are named as inventors on combining siRNA with therapeutic vaccination (PCT/EP2018/028116). UP is a co-founder, shareholder, and board member of SCG Cell Therapy and serves as an *ad hoc* advisor to AAtech, Aligos, Arbutus, Gilead, GSK, Integer Bio, Leukocare, Merck, Sanofi, and Roche. The remaining authors disclose no conflicts.

Please refer to the accompanying ICMJE disclosure forms for further details.
