# Supplementary material for: Long-term antigen reduction does not achieve durable HBV control in mice but enhances therapeutic vaccine efficacy
Source: JHEP Rep. 2026 Jun 30;8(10):101943. doi: 10.1016/j.jhepr.2026.101943 (PMC13524714; doi:10.1016/j.jhepr.2026.101943)
Supplement: Supplementary file 1 [file mmc1.pdf]

Long-term antigen reduction does not achieve durable HBV control in mice but enhances therapeutic vaccine efficacy

Thomas Michler, Anna Kosinska, Osman Merdan, Philipp Hagen, Till Bunse, Edanur Ates Öz, Jinpeng Su, Dirk H. Busch, Carolin Mogler, Ulrike Protzer

Table of contents

Supplementary methods……………………………………………………………….2

Table S1…………………………………………………………………………………….4

Fig. S1……………………………………………………………………………………….4

Fig. S2……………………………………………………………………………………….5

Fig. S3……………………………………………………………………………………….6

Fig. S4……………………………………………………………………………………….7

Fig. S5……………………………………………………………………………………….8

Fig. S6……………………………………………………………………………………….9

Supplementary references……………………………………………………………..9

Supplementary methods

Detection of HBV-specific T cells. Splenocytes and liver-associated lymphocytes were isolated and stained with MHC class I multimers as described[1]. Multimers conjugated with K^b-restricted HBV-peptides $S_{190\text{-}197}$ (S_{190}; VWLSAIWM), $core_{93\text{-}100}$ (C_{93}; MGLKFRQL), MVA-peptide $B8R_{20\text{-}27}$ (MVA_{B8R}; TSYKFESV), and as a control ovalbumin-peptide (SIINFEKL). Per sample, 0.4 µg multimer was labeled with 0.4 µl Streptactin-PE (IBA Lifesciences, Göttingen, Germany) in 30 µl FACS buffer and incubated on ice for 30 min before adding to the cells.

Transcriptome analysis. Liver tissue stored in RNAlater was homogenization using a TissueLyser LT (Qiagen). Total RNA was extracted from 50 mg of liver tissue using the PureLink™ RNA Mini Kit (Invitrogen). Poly(A) enriched (Novogene, Munich, Germany) libraries were sequenced on a NovaSeq X Plus platform (Illumina) using paired-end 150 bp reads (20 million reads per sample). Filtered–trimmed FASTQ sequences were aligned to the GRCm39 (Gencode vM38) genome using the STAR (version 2.7.11b) aligner[2] and gene count matrices generated using featureCounts (version 2.1.1)[3]. Differential gene expression was analyzed using the EdgeR quasi-likelihood pipeline (version 4.8.2, R version 4.5.1)[4] after gene set enrichment analysis using ClusterProfiler (version 4.18.4)[5]. Visualizations were achieved using enrichplot (version 1.3.4), ComplexHeatmap (version 2.26.0), ggplot2 (version 4.0.1), and cytoscape (version 3.10.4)[6]. The sequencing data is accessible via the NCBI's Gene Expression Omnibus[7] and accession number GSE324334.

Histology and immunohistochemistry. Serial 2 µm sections of formalin-fixed, paraffin-embedded liver tissues were stained with hematoxylin-eosin (HE) or rabbit anti-HBV core antibodies (Diagnostic Biosystems, Pleasanton, CA, USA; #RP 017; 1:50 dilution; retrieval at 100°C for 30 min with EDTA) on a Leica Bond MAX system (Leica Biosystems, Nussloch, Germany). Tissue slides were scanned using a SCN400 slide scanner (Leica Biosystems).

Positive cells were quantified from at least 3 tissue areas totaling≥10 mm^2 using the integrated Tissue AI software (Leica Biosystems).

Statistical analyses. Statistical analyses were performed using GraphPad Prism version 9.0. Data sets were initially analyzed for Gaussian distribution with D'Agostino-Pearson omnibus test. Depending on the results, parametric student t test, 1- or 2-way Anova with Tukey´s, Dunnett´s or Sidak´s multiple comparison correction or non-parametric Mann-Whitney U or Kruskal-Wallis with Dunn´s multiple comparison correction tests were used. Linear regression was analyzed using two-tailed Pearson correlation. *p*-values <0.05 were considered significant.

Table S1. Oligonucleotides and cycling conditions used during polymerase chain reaction

Primer name	Primer sequence (5´>3´)	Target	Cyclcing conditions
HBV1464_F	GGACCCCTTCTCGTGTTACA	HBV DNA	2 min 50°C, 10 min 95°C followed by 45× (15 s 95°C, 1 min 60°C)
HBV1599_R	ACTGCGAATTTTGGCCAAGA		
HBV1529_P	FAM-TCTAGACTCGTGGTGGACTT CTCTCAATTTTCT-TAMRA		
HBV3.5kbRNA_F	GAGTGTGGATTCGCACTCC	HBV 3.5 kb RNA	5 min 95°C followed by 40× (3 s 95°C, 30 s 60°C)
HBV3.5kbRNA_R	GAGGCGAGGGAGTTCTTCT		
mGAPDH_F	ACCAACTGCTTAGCCC	Murine *gapdh* mRNA	5 min 95°C followed by 40× (15 s 95°C, 10 s 60°C, 25 s 72°C)
mGAPDH_R	CCACGACGGACACATT		
AAV_ITR_F	AACCCGCCATGCTACTTATCTACCT	AAV inverse terminal repeat	5 min 95°C followed by 40× (15 s 95°C, 10 s 60°C, 25 s 72°C)
AAV_ITR_R	CAAACAGTCTTTGAAGTATGCC		
mPrp_F	GCGGTACATGTTTTCACGGTAGTA	Murine *prion protein* gene	5 min 95°C followed by 40× (15 s 95°C, 10 s 60°C, 25 s 72°C)
mPrp_R	GAGCAGGCCCATGATCCA		

Table S2. List of differentially expressed genes from bulk sequencing analysis

Table S3. Results from gene set enrichment analysis. List contains all pathways that were significantly de-regulated between the shCtrl and shHBV 8 week groups.

Fig. S1

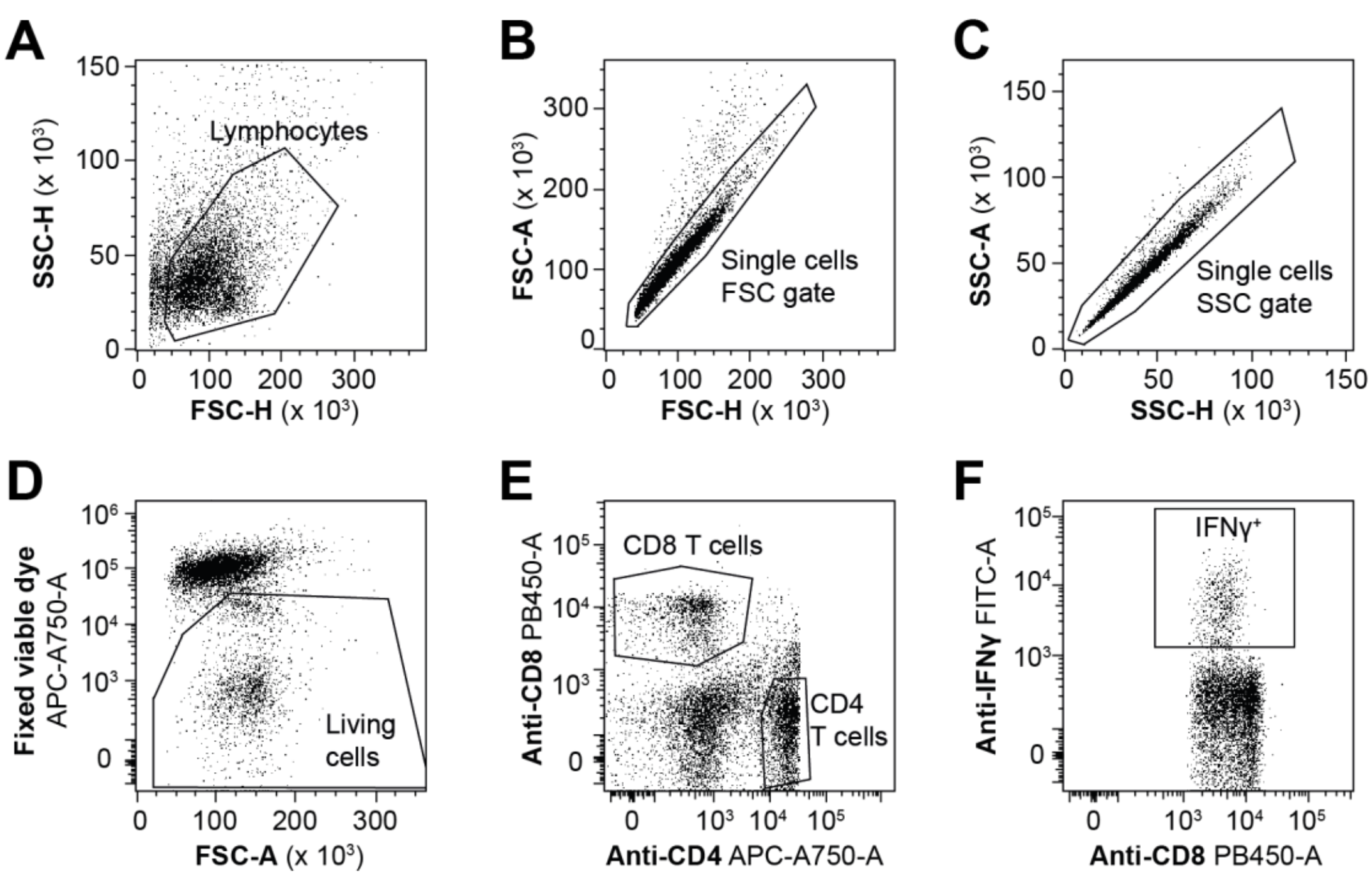

Fig. S1. Flow cytometry gating strategy to quantify HBV-reactive CD8 T cells. Liver associated lymphocytes were isolated from livers of mice and re-stimulated *ex vivo* with HBV-specific peptide pools. After gating on lymphocytes (**A**), cell clumbs or dublets were excluded using FSC- (**B**) as well as SSC- (**C**) based single cell gates. (**D**) Dead cells were excluded using a Fixed viable dye. (**E**) Gating employed to identify $CD8^+$ and $CD4^+$ T cells and (**F**) Interferon gamma (IFNγ) positive CD8 T cells. FSC, forward scatter; SSC, sideward scatter. H, height; A, Area.

Fig. S2

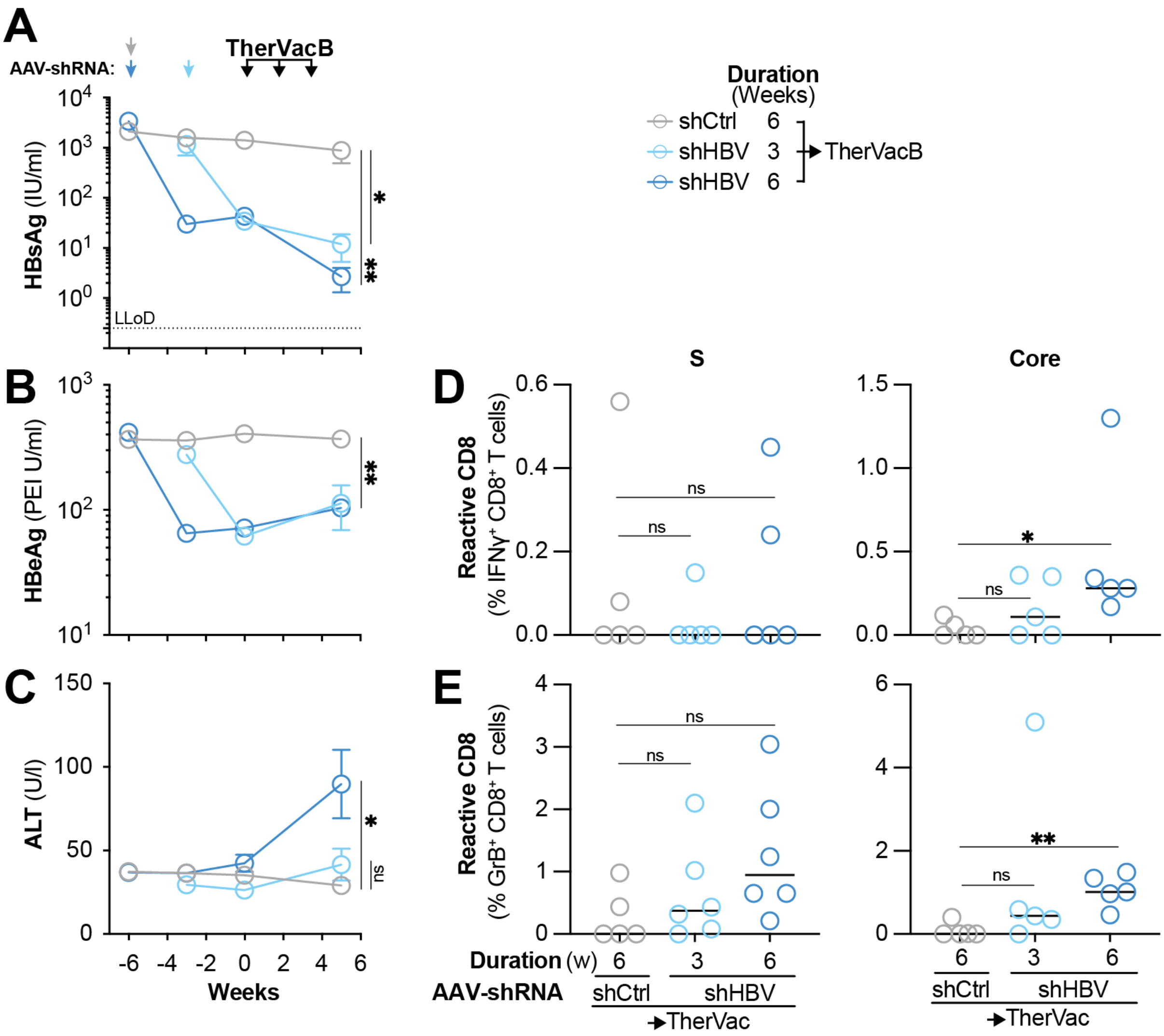

Fig. S2. Influence of the duration of HBV antigen suppression by an shRNA-expressing AAV vector on efficacy of therapeutic vaccination. HBV-transgenic mice were injected intravenously with 10^{11} genome equivalents of an AAV vector expressing an HBV-specific shRNA (AAV-shHBV) or a control shRNA (shCtrl). Mice were immunized with the *TherVacB* regimen starting either 3 or 6 weeks after injection of the AAV vector, and the effect on HBV replication and HBV-specific immunity was analyzed. (**A**, **B**) Serum levels of (**A**) HBsAg and (**B**) HBeAg were determined using chemiluminescence immunoassays. (**C**) The activity of Alanine Aminotransferase (ALT) was measured using a chromogenic assay. (**D**, **E**) Intrahepatic HBV-specific CD8 T cell responses were determined following *ex vivo* re-stimulation with an HBs-derived (S_{190}, left graphs) or HBc-derived (C_{93}, right graphs) peptides by intracellular staining for (**D**) IFNγ or (**E**) granzyme B (GrB). Experimental groups contained 5 animals each. In panels A-C, the mean and standard error of the mean, in panels D and E, the median is indicated. Statistical differences were calculated using the Kruskal-Wallis test with multiple comparison corrections. ns, LLoD, lower limit of detection; non-significant; *****, p<0.05; ******, p<0.01

Fig. S3

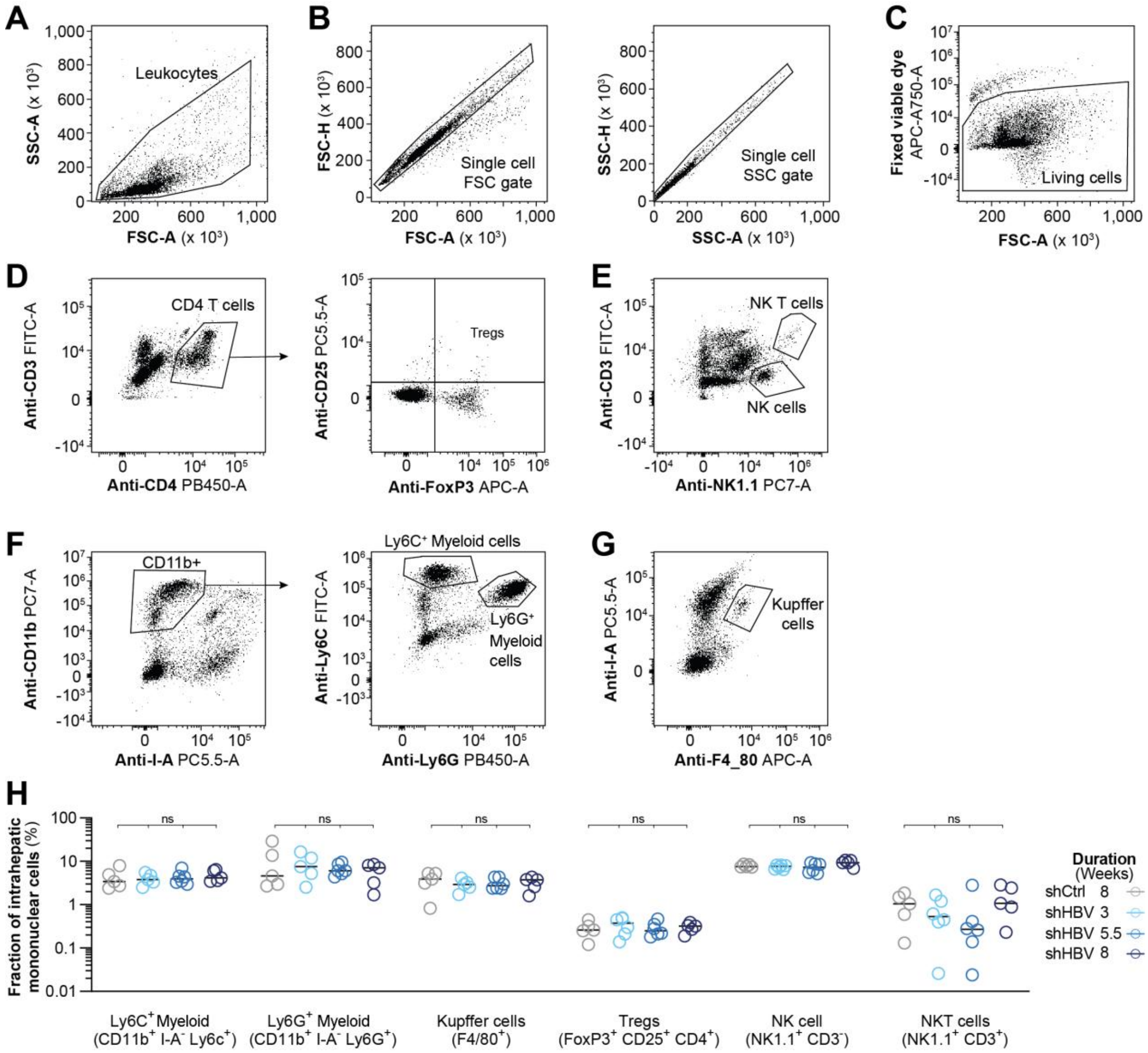

Fig. S3. Influence of the duration of RNAi treatment on the distribution of intrahepatic immune cell populations. HBV-transgenic mice (HBVxfs line) were treated intravenously at varying time points (3, 5.5 or 8 weeks before the end of the experiment) with 10^{11} genome equivalents of an Adeno-Associated Virus (AAV) vector that expresses an HBV-specific short-hairpin (shHBV) or a control shRNA (shCtrl). Immune cells were isolated from livers of mice and quantified by FACS. (**A**-**G**) Gating strategies employed to quantify the respective immune cell population. After gating on leukocytes (**A**), cell clumbs or dublets were excluded using (**B**) FSC- as well as SSC-based single cell gates. (**C**) Dead cells were excluded using a Fixed viable dye. (**D**) Regulatory T cells (Tregs) were identified by staining CD4, CD25 and FoxP3, (**E**) NK and NK T cells by staining for NK1.1 and CD3, (**F**) Myeloid cells by staining for CD11b, Ly6C and Ly6G, and (**G**) Kupffer cells by staining I-A and F4_80. FSC, forward scatter; SSC, sideward scatter. H, height; A, Area; Experimental groups contained 5 (shCtrl, shHBV 3W, shHBV 5.5W) or 6 (shHBV 8W) animals. Bars indicate median value. Statistical differences were calculated using One-Way Anova with multiple comparison correction. ns, non-significant

Fig. S4

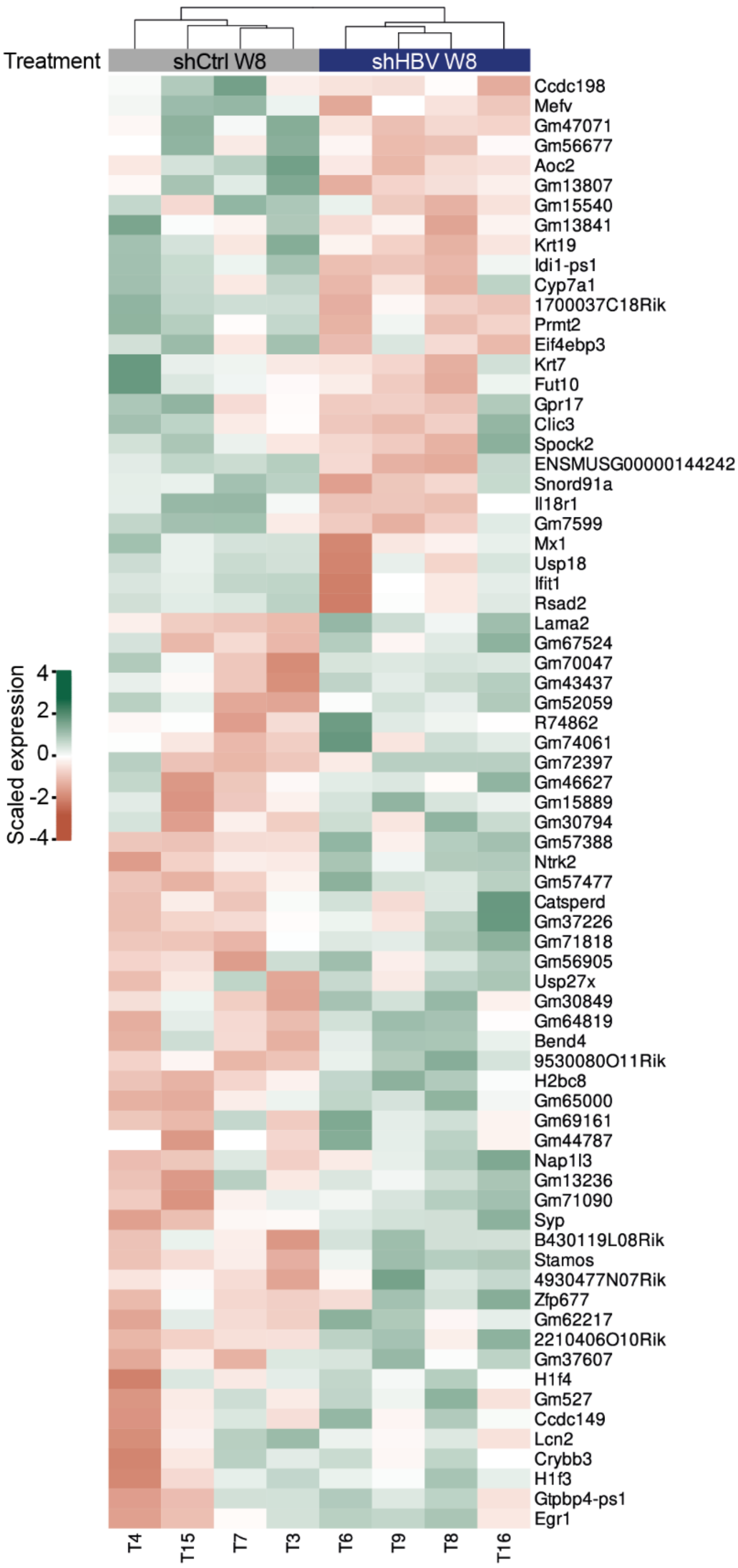

Fig. S4. Influence of RNAi-mediated HBV antigen knockdown for 8 weeks on intrahepatic gene expression. HBV-transgenic mice (HBVxfs line) were treated intravenously at varying time points (3, 5.5 or 8 weeks before the end of the experiment) with 10^{11} genome equivalents of an Adeno-Associated Virus (AAV) vector that expresses an HBV-specific short-hairpin (shHBV) or a control shRNA (shCtrl). RNA was extracted from whole liver lysate of 4 animals per group and after cDNA synthesis bulk sequenced on the Illumina NovaSeq X Plus platform. Heatmaps shows differentially expressed genes (adjusted p <0.05; LogFC >1) between groups of mice treated for 8 weeks with either shHBV or shCtrl.

Fig. S5

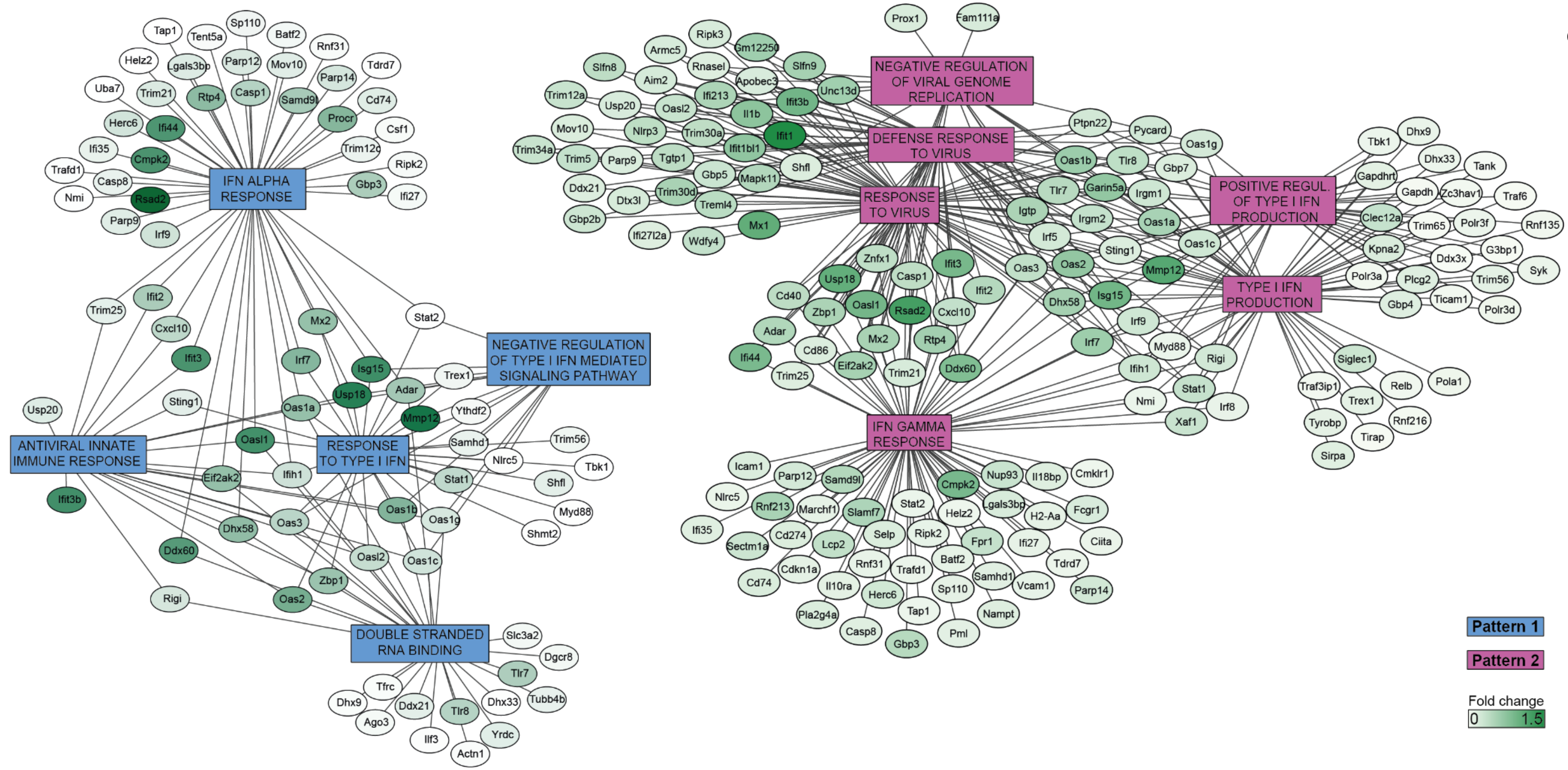

Fig. S5. Influence of the duration of RNAi treatment on intrahepatic signaling networks. HBV-transgenic mice (HBVxfs line) were treated at varying time points (8, 5.5, or 3 weeks before the end of the experiment) intravenously with 10^{11} genome equivalents of an AAV vector expressing an HBV-specific (shHBV) or a control shRNA (shCtrl). RNA of 4 animals per treatment group was extracted and bulk sequenced. Signaling networks of which the regulation correlated with the duration of RNAi treatement (details shown in Figure 4) were analysed by Gene Set Enrichment Analysis and visualized using the Cytoscape software.

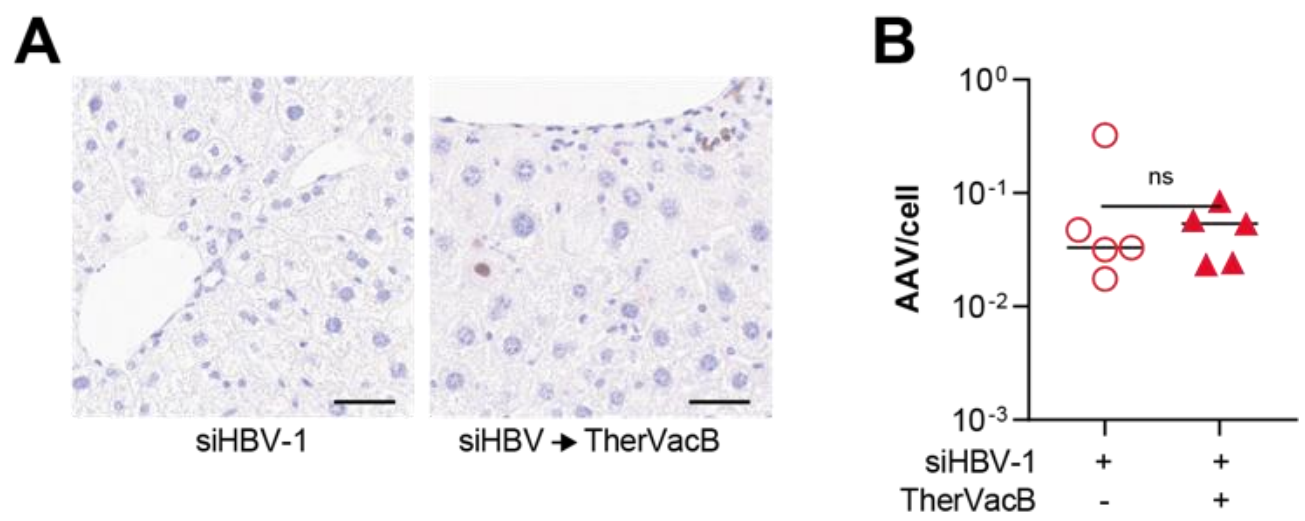

Fig. S6. Influence of therapeutic vaccination on hepatocyte turnover and AAV genome levels in livers of low HBV-carrier mice treated with HBV siRNA. C57/BL6 mice were infected with $8x10^8$ geq of AAV-HBV (same animals as shown in Figure 7). After four weeks, mice were treated with four monthly s.c. injections of 3 mg/kg body weight of an HBV-specific siRNA (siHBV-1). Half of the mice were immunized starting with the last siRNA application, whereas the other mice received no further treatment. The experiment was terminated 23 weeks after start of TherVacB and (**A**) liver sections stained with ki67-specific antibodies and (**B**) AAV genome levels were quantified from liver lysates by quantitative PCR relative to the single copy *prion protein*. Each experimental group contained 5 mice. Bars in (A) indicate 100 µm and in (B) the median. Statistical differences were calculated by Mann Whitney U-Test. Ns, non-significant

Supplementary references

[1] Backes S, Jager C, Dembek CJ, et al. Protein-prime/modified vaccinia virus Ankara vector-boost vaccination overcomes tolerance in high-antigenemic HBV-transgenic mice. Vaccine. 2016;34:923-932.

[2] Dobin A, Davis CA, Schlesinger F, et al. STAR: ultrafast universal RNA-seq aligner. Bioinformatics. 2013;29:15-21.

[3] Liao Y, Smyth GK, Shi W. featureCounts: an efficient general purpose program for assigning sequence reads to genomic features. Bioinformatics. 2014;30:923-930.

[4] Chen Y, Chen L, Lun ATL, et al. edgeR v4: powerful differential analysis of sequencing data with expanded functionality and improved support for small counts and larger datasets. Nucleic Acids Res. 2025;53.

[5] Yu G, Wang LG, Han Y, et al. clusterProfiler: an R package for comparing biological themes among gene clusters. OMICS. 2012;16:284-287.

[6] Shannon P, Markiel A, Ozier O, et al. Cytoscape: a software environment for integrated models of biomolecular interaction networks. Genome Res. 2003;13:2498-2504.

[7] Edgar R, Domrachev M, Lash AE. Gene Expression Omnibus: NCBI gene expression and hybridization array data repository. Nucleic Acids Res. 2002;30:207-210.
