# Supplementary material for: Long-term antigen reduction does not achieve durable HBV control in mice but enhances therapeutic vaccine efficacy
Source: JHEP Rep. 2026 Jun 30;8(10):101943. doi: 10.1016/j.jhepr.2026.101943 (PMC13524714; doi:10.1016/j.jhepr.2026.101943)
Supplement: Supplementary file 4 [file mmc2.docx]

**JHEP Reports**

**CTAT methods**

Tables for a “Complete, Transparent, Accurate and Timely account” (CTAT) are now mandatory for all revised submissions. The aim is to enhance the reproducibility of methods.

- Only include the parts relevant to your study
- Refer to the CTAT in the main text as ‘Supplementary CTAT Table’
- Do not add subheadings
- Add as many rows as needed to include all information
- Only include one item per row

**If the CTAT form is not relevant to your study, please outline the reasons why:**

1.1 **Antibodies**

| **Name** | **Citation** | **Supplier** | **Cat no.** | **Clone no.** |
| --- | --- | --- | --- | --- |
| anti-mCD8 PB |  | BD Biosciences, Heidelberg, Germany | 558106 | 56.6-7 |
| anti-mCD4 APC |  | eBioscience, Thermo Fischer Scientific | 17-0041-84 | L3T4 |
| anti-mIFNγ FITC |  | eBioscience, Thermo Fischer Scientific | 554411 | XMG1.2 |
| anti-mTNF PE-Cy7 |  | BD Biosciences | 557644 | MP6-XT22 |

1.2 **Cell lines**

| **Name** | **Citation** | **Supplier** | **Cat no.** | **Passage no.** | **Authentication test method** |
| --- | --- | --- | --- | --- | --- |
| Not applicable |  |  |  |  |  |

1.3 **Organisms**

| **Name** | **Citation** | **Supplier** | **Strain** | **Sex** | **Age** | **Overall n number** |
| --- | --- | --- | --- | --- | --- | --- |
| Mus musculus | Dumortier, J. *et al.* Liver-specific expression of interferon gamma following adenoviral gene transfer controls hepatitis B virus replication in mice. *Gene Ther* **12**, 668-677, doi:10.1038/sj.gt.3302449 (2005) | Local breeding | HBVxfs | male | 8-12 weeks | 81 |
| Mus musculus | Not available | Charles River laboratories | C57BL/6J | male | 8-12 weeks | 22 |

1.4 **Sequence based reagents**

| Name | **Sequence** | **Supplier** |
| --- | --- | --- |
| S190-197 (S190) | VWLSAIWM | peptides & elephants GmbH, Germany |
| Core 93-100 (C93) | MGLKFRQL | peptides & elephants GmbH, Germany |
| MVA-derived peptide B8R20-27 (MVAB8R) | TSYKFESV | peptides & elephants GmbH, Germany |
| Ovalbumin-derived peptide S8L 257-264 (OVAS8L) | SIINFEK | peptides & elephants GmbH, Germany |
| S208-215 (S208) | IVSPFIPL | peptides & elephants GmbH, Germany |

1.5 **Biological samples**

| **Description** | **Source** | **Identifier** |
| --- | --- | --- |
| Not applicable |  |  |

1.6 **Deposited data**

| **Name of repository** | **Identifier** | **Link** |
| --- | --- | --- |
| NCBI's Gene Expression Omnibus | accession number GSE324334 | [https://www.ncbi.nlm.nih.gov/geo/query/acc.cgi?acc=GSE324334](https://www.ncbi.nlm.nih.gov/geo/query/acc.cgi?acc=GSExxx) |

1.7 **Software**

| **Software name** | **Manufacturer** | **Version** |
| --- | --- | --- |
| STAR aligner | Alexander Dobin | version 2.7.11b |
| featureCounts | Academic; for details see publication: <https://doi.org/10.1093/bioinformatics/btt656> | version 2.1.1 |
| EdgeR quasi-likelihood pipeline | Academic; for details see publication:  <https://doi.org/10.1093/nar/gkaf018> | version 4.5.1 |
| ClusterProfiler^5^ | Academic; for details see publication:  https://doi.org/10.1089/omi.2011.0118 | version 4.18.4 |
| enrichplot | Academic; for details see publication:  https://doi.org/[10.18129/B9.bioc.enrichplot](https://doi.org/doi:10.18129/B9.bioc.enrichplot) | version 1.3.4 |
| ComplexHeatmap | Academic; for details see publication:  https://doi.org/[10.18129/B9.bioc.ComplexHeatmap](https://doi.org/10.18129/B9.bioc.ComplexHeatmap) | version 2.26.0 |
| ggplot2 | Academic; for details see publication:  https://doi.org/[10.32614/CRAN.package.ggplot2](https://doi.org/10.32614/CRAN.package.ggplot2) | version 4.0.1 |
| cytoscape | Academic; for details see publication:  https://doi.org/[10.1101/gr.1239303](https://doi.org/10.1101/gr.1239303) | version 3.10.4 |

1.8 **Other (*e.g*. drugs, proteins, vectors etc.)**

| Not applicable |  |  |
| --- | --- | --- |

1.9 **Please provide the details of the corresponding methods author for the manuscript:**

Prof. Ulrike Protzer, protzer@tum.de

**2.0	Please confirm for randomised controlled trials all versions of the clinical protocol are included in the submission. These will be published online as supplementary information.**

Not applicable
